# Stimulus-Responsive Polymeric Carriers for Gene Delivery: Balancing Endosomal Escape with Nucleic Acid Release

**DOI:** 10.1007/s11095-026-04173-6

**Published:** 2026-08-07

**Authors:** Kenneth Hulugalla, Alexander W. Fortenberry, Dilusha J. de Silva, Thomas Werfel

**Affiliations:** 1 Department of BioMedical Engineering, University of Mississippi, University, MS 38677 USA; 2 Department of BioMolecular Sciences, University of Mississippi, University, MS 38677 USA; 3 Department of Chemical Engineering, University of Mississippi, University, MS 38677 USA; 4 Cancer Center and Research Institute, University of Mississippi Medical Center, Jackson, MS 39216 USA

**Keywords:** endosomal escape, genetic medicine delivery, nucleic acid release, polymeric carriers, stimulus-responsive polymers

## Abstract

**Background:**

Polymeric carriers for nucleic acid delivery must overcome two sequential intracellular barriers: escape from the endosome and release of the cargo into the cytosol. These steps are typically treated as independent design problems and optimized separately.

**Objective:**

This review argues that escape and release are coupled through the same electrostatic and pH-dependent interactions, creating a fundamental tradeoff . The cationic charge that enables a polymer to destabilize endosomal membranes also tightens its grip on the nucleic acid payload, so conditions favoring escape simultaneously inhibit release.

**Methods:**

We trace this tradeoff across the major strategy classes in the polymeric delivery literature, evaluating each by where and when its trigger acts relative to the escape event. We further assess the assay toolkit for measuring escape and release, and the translational variables that shift the balance *in vivo*.

**Results:**

pH-responsive polymers tune the activation zone for membrane destabilization but cannot decouple escape from binding, since both depend on the same protonation events. Charge-shifting polymers address release more directly by programming a loss of cationic character after endosomal entry. Non-pH triggers, including disulfide, thioketal, diselenide, and esterase-responsive chemistries, off er orthogonal release mechanisms but face spatial and temporal alignment constraints. Characteristic failure modes recur: premature release, redundant triggering, and kinetic misalignment. No existing assay measures endosomal disruption and cargo release state simultaneously, and translational variables including protein corona formation, cell-type-dependent endosomal acidifi cation, and PEG shielding shift the balance in ways *in vitro* screening does not capture.

**Conclusion:**

The tradeoff cannot be resolved by chemistry alone, meaning the responsive trigger considered in isolation. Its resolution requires coordination across spatial (architectural domain segregation), temporal (kinetic alignment of escape and release), and formulation-level (N/P ratio, PEG density, formation pH) design axes.

**Graphical Abstract:**

**Figure:**
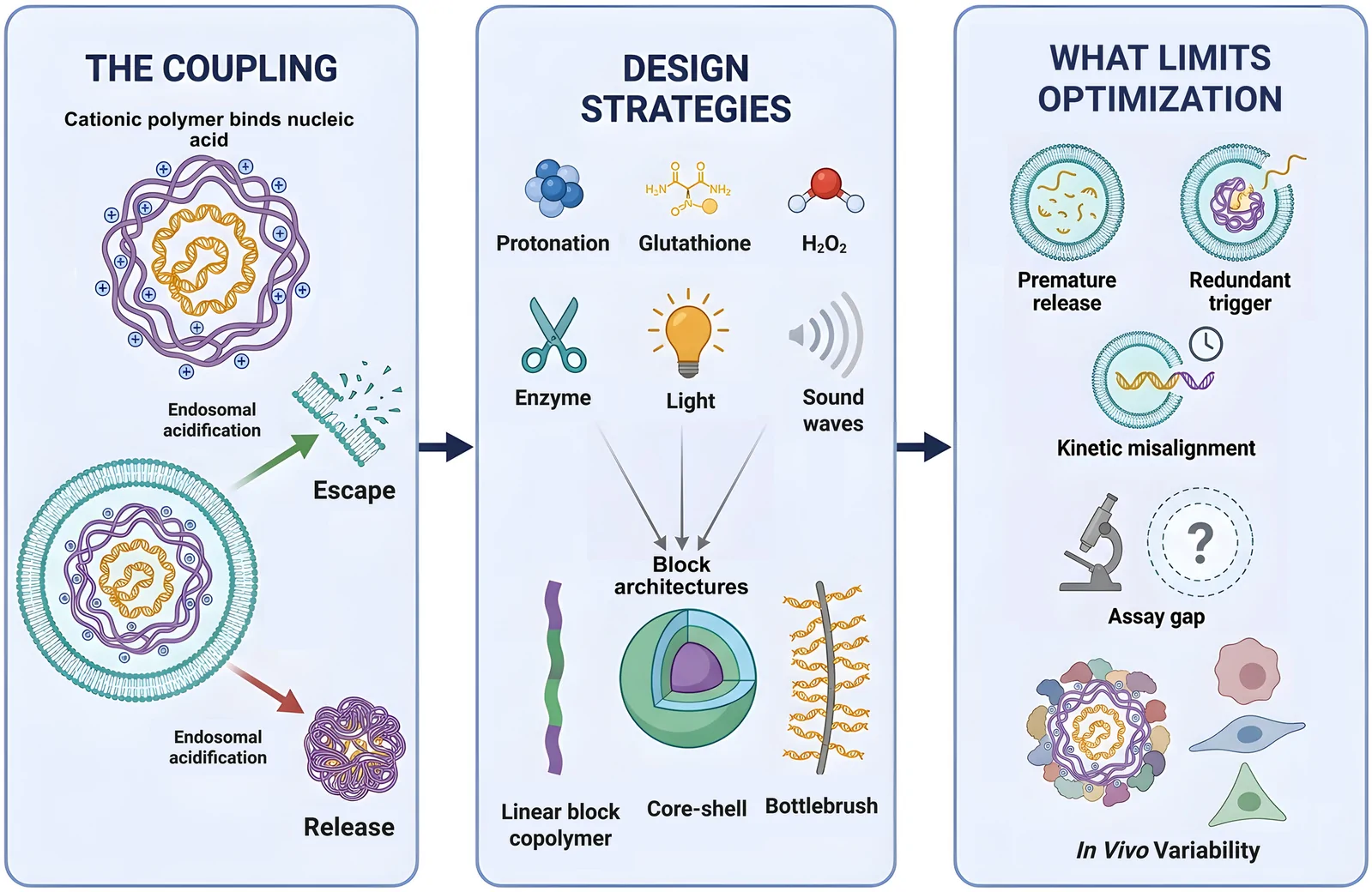

## Introduction

Gene-based medicines have shifted the therapeutic landscape from modulating proteins after they are made to controlling the flow of biological information itself. Small interfering RNA (siRNA), antisense oligonucleotides (ASO), messenger RNA (mRNA), plasmid DNA, self-amplifying RNA, circular RNA, and CRISPR-associated nucleic acid systems can silence disease genes, restore missing proteins, transiently reprogram cells, express antigens, or introduce targeted genetic edits [1–4]. This breadth of function explains the intense interest in nucleic acid therapeutics. Yet their clinical impact depends on solving a difficult delivery problem. Nucleic acids are large, hydrophilic, polyanionic macromolecules that are vulnerable to nuclease degradation, inefficient at crossing cellular membranes, and prone to sequestration within endosomal and lysosomal compartments after uptake [1, 5, 6]. For genetic medicines, therapeutic success depends not only on the payload itself, but on the carrier system that protects it, transports it, and releases it at the correct intracellular site. It should be noted that some ASO chemistries, such as phosphorodiamidate morpholino oligomers (PMOs) and peptide nucleic acids (PNAs), lack the negatively charged phosphodiester backbone and therefore cannot form electrostatic polyplexes [7, 8]. The escape–release tradeoff as defined in this review applies specifically to polyanionic nucleic acids that complex with cationic carriers through charge-based interactions.

The clinical success of lipid nanoparticles (LNPs), including the first approved siRNA therapeutic and the mRNA vaccines deployed during the COVID-19 pandemic, has demonstrated that synthetic nonviral carriers can overcome enough of these barriers to produce meaningful human benefit [3, 9]. However, LNPs still face limitations in extrahepatic targeting, cytosolic release efficiency, and repeated-dosing tolerability that constrain their utility beyond vaccination and liver-directed therapy [5, 10–12]. Quantitative studies estimate that fewer than 2% of LNP-encapsulated nucleic acids reach the cytosol after uptake, with the remainder degraded in endolysosomal compartments or recycled out of the cell [13, 14]. LNPs also preferentially accumulate in the liver through apolipoprotein E-mediated uptake, and redirecting tropism to non-hepatic tissues without sacrificing delivery efficiency remains an active challenge [15]. For applications requiring repeated administration, anti-PEG immune responses can reduce efficacy over successive doses [16]. These constraints are particularly limiting for protein replacement, immune cell engineering, and genome editing, where tissue selectivity, controllable intracellular release, and durable dosing are essential.

This review focuses on polymer-based carriers, referred to hereafter as polyplexes, which are structurally and mechanistically distinct from LNPs and from polymer-lipid hybrids. Polyplexes form through electrostatic complexation between cationic polymers and anionic nucleic acids, producing self-assembled particles held together by charge–charge interactions [1, 17]. LNPs, by contrast, encapsulate nucleic acid within a matrix of ionizable lipid, cholesterol, helper phospholipid, and PEG-lipid, and their endosomal escape depends on protonation of the ionizable lipid at low pH followed by membrane fusion or destabilization, rather than on a shared electrostatic backbone with the cargo [13, 18]. Polymer-lipid hybrids, including lipopolyplexes and lipid-coated polyplexes, retain a polyplex core surrounded by a lipid shell and combine features of both architectures [19]. All three systems must cross an endosomal membrane and release cargo in a functional form, but the coupling between binding, escape, and release differs by architecture. The escape–release tradeoff developed here is most acute for simple polyplexes; where LNP or hybrid data are used to support general principles, we note the mechanistic differences that shape interpretation.

Polymeric carriers offer a complementary design space. Unlike small-molecule lipids, synthetic polymers can be tuned across multiple structural levels: monomer chemistry, molecular weight, charge density, hydrophobicity, degradability, sequence, topology, architecture, and ligand display [1, 4, 17]. Cationic polymers such as polyethylenimine, poly(L-lysine), poly(amidoamine) dendrimers, poly(β-amino esters), poly(amino acids), poly(aspartamides), and charge-altering releasable transporters form electrostatic complexes with anionic nucleic acids (polyplexes) that can condense cargo into nanoscale particles, protect it from degradation, promote uptake, and participate in endosomal membrane disruption [4, 17, 20–22]. The synthetic flexibility of polymers also allows incorporation of stimuli-responsive motifs that change charge, hydrophobicity, solubility, or degradability in response to biological cues such as pH, redox potential, reactive oxygen species, or enzymes [2, 23, 24].

This tunability is the major promise of polymeric delivery, but it also reveals the central difficulty. The optimal carrier property changes at each step of the intracellular pathway, and the properties that promote one step often oppose the next. A carrier must bind tightly enough to protect its cargo through uptake and trafficking, acquire enough membrane activity to escape the endosome, and then release the nucleic acid in a biologically accessible form. The field has traditionally emphasized endosomal escape as the dominant intracellular bottleneck, and for good reason: most internalized nanoparticles remain trapped in endosomal or lysosomal compartments [10–12, 25]. However, escape alone is not equivalent to functional delivery. A nucleic acid that exits the endosome while still tightly complexed to its carrier may be physically present in the cytosol but biologically unavailable [26–30].

This review is organized around a design conflict specific to polymeric nucleic acid carriers. Unlike LNPs, whose escape and release functions are distributed across separate lipid components, polymeric carriers must combine cargo condensation and endosomal membrane activity within the same chemical structure. The consequences of this constraint for carrier design are traced through the following sections, culminating in the argument that endosomal escape and nucleic acid release cannot be separately optimized without addressing the coupling that arises from this shared structural role [20, 21, 31]. This escape–release tradeoff is not a minor formulation detail. It is a fundamental design conflict that helps explain why many polymeric carriers perform well in uptake or escape assays but fail in functional delivery.

Stimulus-responsive polymers are best understood as attempts to manage this conflict. pH-responsive polymers tune where membrane activity begins [17, 22, 32]. Charge-shifting systems weaken electrostatic binding after entry by converting cationic groups to neutral or anionic states [2, 33, 34]. Redox-, ROS-, and enzyme-responsive systems introduce triggers orthogonal to pH [23, 24, 35]. Dual-trigger and sequential-trigger systems attempt to coordinate multiple transitions across the delivery pathway. However, stimulus responsiveness does not automatically solve the delivery problem. A trigger must occur in the right compartment, at the right time, and with the right kinetics for the intended cargo. As this review will argue, no single chemical trigger, on its own, has yet resolved the escape–release tradeoff. The central question is not whether a polymer is "responsive," but whether the response is synchronized with the biological sequence of delivery.

This challenge is compounded by the diversity of nucleic acid cargos, each with different size, topology, intracellular destination, and tolerance for carrier association [3, 4, 36], and by limitations in the current assay toolkit, which can typically measure either endosomal disruption or functional activity, but not the complexation state of the cargo at the moment of escape [25, 28–30, 37, 38].

This review evaluates stimulus-responsive polymeric carriers through the lens of the escape–release tradeoff. We first define its basis, emphasizing how protonation, charge density, and membrane activity are linked to cargo binding (Sect. "The Mechanistic Basis of the Escape–Release Tradeoff"). We then examine why the tradeoff shifts with cargo type (Sect. "Cargo-Specific Considerations: Why One Size Does Not Fit All") and assess pH-responsive and charge-shifting strategies (Sect. "pH-responsive and charge-shifting polymers as escape–release coupling devices"), followed by non-pH triggers including disulfides, thioketals, diselenides, and esterase-sensitive systems (Sect. "Non-pH Stimulus-Responsive Strategies: Do Alternative Triggers Resolve the Tradeoff?"). We consider how polymer architecture and formulation can physically separate escape and release functions (Sect. "Polymer Architecture and Formulation: Structural Approaches to Decoupling Escape from Release"), discuss assay limitations (Sect. "Measuring Escape and Release: The Assay Gap"), and evaluate translational variables that shift the balance *in vivo* (Sect. "Translational Considerations: Why the Tradeoff Is Harder *In Vivo*"). Our goal is to move beyond cataloging responsive chemistries and toward a design framework based on timed state transitions: protect extracellularly, activate endosomally, escape efficiently, and release cargo in a biologically active form.

## The Mechanistic Basis of the Escape–Release Tradeoff

Endosomal escape is widely recognized as the rate-limiting step in polymeric nucleic acid delivery, yet escape alone does not guarantee therapeutic activity [39]. Once in the cytosol, nucleic acid cargo must dissociate from its polymeric carrier to engage cellular machinery: siRNA must load into the RNA-induced silencing complex (RISC), mRNA must access ribosomes, and plasmid DNA must traffic to the nucleus [40, 41]. The physicochemical properties that promote escape (high cationic charge density, strong electrostatic binding, membrane-disruptive capacity) are the same properties that inhibit cargo release. Most existing reviews treat escape and release as separate optimization problems. Here, we argue that they are coupled through the same electrostatic and pH-dependent interactions, and that this coupling is the central design challenge for polymeric nucleic acid carriers [13, 39, 42].

### How Polymeric Escape Mechanisms Create the Release Problem

**Fig. 1:**
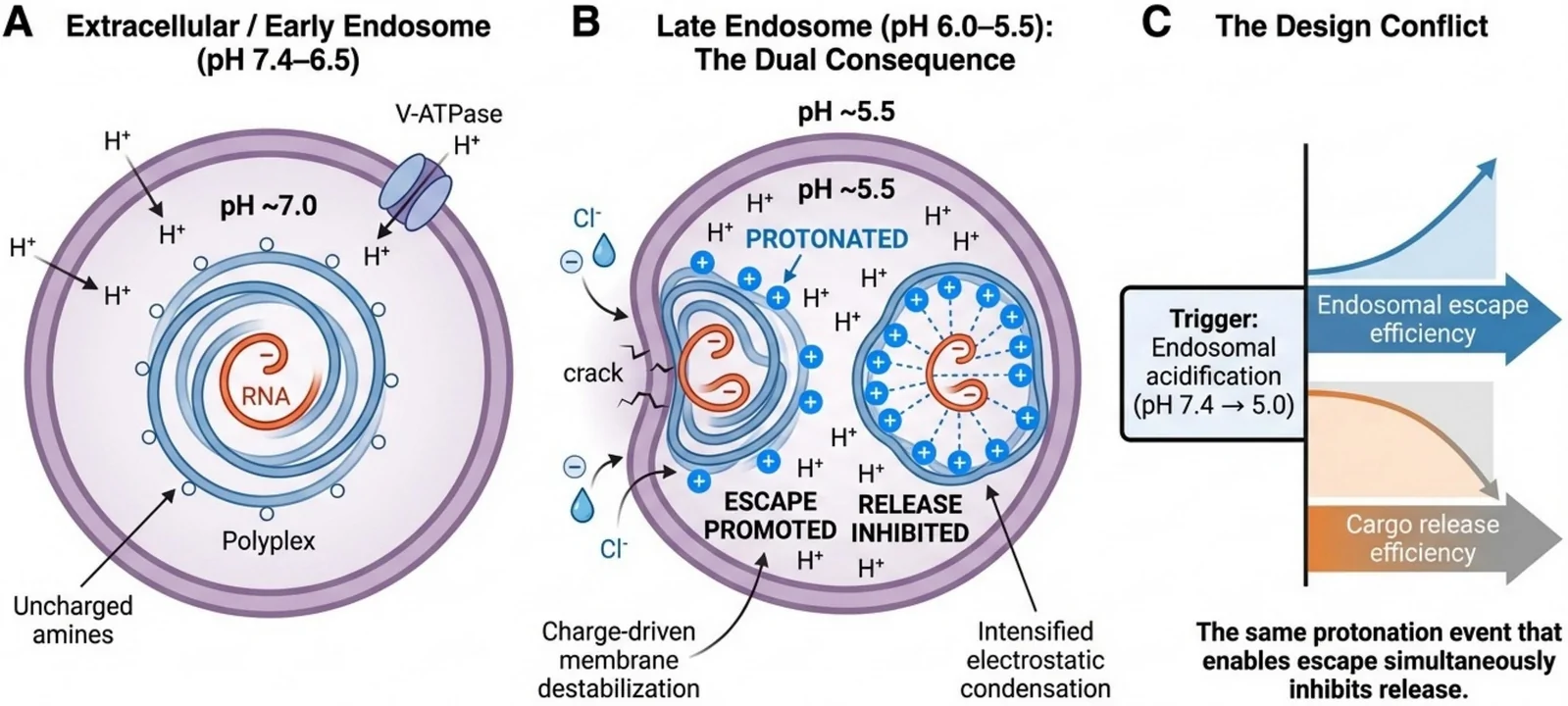
The dual consequence of endosomal acidification for polymeric nucleic acid carriers. (**A**) At physiological pH, amine groups on the polymer backbone are largely uncharged. (**B**) Endosomal acidification protonates these amines, simultaneously driving membrane (promoting escape) and tightening electrostatic condensation of the nucleic acid cargo (inhibiting release). (**C**) This mechanistic coupling creates a fundamental design conflict: conditions that favor escape inhibit release.

The dominant escape mechanisms proposed for polymeric carriers (proton buffering with osmotic stress, and direct membrane destabilization by cationic charge) have been extensively reviewed elsewhere [38, 39, 43, 44]. What has received far less attention is how these mechanisms couple to cargo binding, the question this section examines. This dual consequence of a single chemical trigger is the origin of the escape–release tradeoff (Fig. 1).

In the proton sponge model, polymers such as polyethylenimine (PEI) and poly(amidoamine) (PAMAM) dendrimers buffer endosomal acidification, driving chloride and water influx that contributes to membrane stress [45, 46]. Whether osmotic pressure alone can rupture endosomes remains debated. Direct pH measurements suggest PEI does not substantially alter endosomal pH in living cells, and modeling indicates that osmotic contributions may be modest relative to the mechanical strength of the endosomal membrane [31, 47]. The current consensus favors a combined mechanism in which polyplex swelling upon protonation, electrostatic association of cationized polymer with anionic endosomal membrane lipids, and osmotic effects act together to destabilize membrane integrity [47]. The critical point for this review is not which of these sub-mechanisms dominates, but that all three are activated by the same event: protonation of polymer amines. That same event also increases the charge density holding the nucleic acid cargo in place. A polymer cannot become more cationic to disrupt the endosomal membrane without simultaneously becoming more cationic toward its own cargo.

Direct membrane destabilization follows the same logic. As endosomal pH drops, progressive protonation increases the net positive charge on free and polyplex-associated polymer, enabling electrostatic interaction with anionic phospholipids (particularly bis(monoacylglycero)phosphate, enriched on the inner leaflet of late endosomal membranes) and inducing local pore formation [38]. The polymer's membrane-disruptive potency scales with its degree of protonation, but so does its grip on the polyanionic nucleic acid backbone [48, 49].

The most revealing data on this interface comes from live-cell imaging by Rehman and colleagues, who tracked fluorescently labeled polymer and nucleic acid separately during polyplex-mediated delivery [29]. Their observations deserve detailed consideration because they directly illuminate how escape and release interact. First, productive endosomal release was a rare event, originating from only one or two endosomes per cell out of the hundreds to thousands that contained internalized polyplexes. Well over 99% of internalized particles never contributed to functional delivery. Second, at the moment of escape, polymer and nucleic acid were separately expelled through what appeared to be local pores in the endosomal membrane, rather than released as intact polyplexes through wholesale endosomal rupture. Third, this separation occurred rapidly, suggesting that the physical forces during pore-mediated expulsion (shear, concentration gradients, or interaction with membrane lipids) may contribute to dissociation in ways that bulk decomplexation assays conducted in solution do not capture [29].

These observations raise questions that the field has not adequately addressed. If polymer and nucleic acid separate during pore-mediated escape, it is an open question whether the nucleic acid entering the cytosol is genuinely free or is transferred onto cytosolic polycations or membrane-associated proteins. Endogenous mRNA and siRNA traffic through the cytosol and engage ribosomes or RISC efficiently, so this concern applies specifically to carrier-bound cargo whose release from polymer may be incomplete, not to free nucleic acid, which has no comparable navigational barrier. The relevant question is therefore kinetic rather than thermodynamic: whether cargo is handed off to translation or silencing machinery faster than it is re-bound by competing cytosolic polycations, not whether re-binding is thermodynamically permissible. It is equally unclear whether the escaped fraction is biased toward loosely bound cargo at the polyplex periphery, or whether the transient pore size imposes a physical filter that discriminates between free nucleic acid and intact polyplexes. These dynamics are likely cargo-dependent, given that siRNA (~ 14 kDa, 42 phosphate groups) and mRNA (300–1500 kDa, thousands of phosphate groups) differ by orders of magnitude in both their electrostatic contacts and hydrodynamic diameter (Sect. "Cargo-Specific Considerations: Why One Size Does Not Fit All"). Resolving these questions will require the improved assay capabilities discussed in Sect. "Measuring Escape and Release: The Assay Gap", which we argue must be able to distinguish escaped-but-bound nucleic acid from escaped-and-free nucleic acid.

### Why Escape and Release Are Inherently Opposed

Endosomal escape demands high cationic charge to destabilize membranes. Cargo release demands weakened cation–anion interactions to liberate nucleic acids. In polymeric systems, these two requirements are tied to the same pH gradient, creating a design conflict that cannot be resolved by optimizing charge alone.

#### Electrostatic Binding Intensifies with Endosomal Acidification

The relationship between pH and binding strength is governed by the pKₐ of the polymer's ionizable groups. For polymers with pKₐ values well above 7.4, such as most PEI variants (pKₐ ~ 8–10 for primary amines, ~ 6–7 for secondary and tertiary amines across the branched structure) and poly(L-lysine) (PLys, pKₐ ~ 10 for ε-amino groups), the majority of ionizable groups are already protonated at physiological pH, and endosomal acidification increases charge further [50, 51]. However, the reported pKₐ values for PEI represent bulk averages across a heterogeneous population of amines. In practice, electrostatic repulsion between neighboring protonated amines in a densely branched architecture suppresses further protonation of adjacent groups, meaning that a significant fraction of amines remain unprotonated even at pH values well below their nominal pKₐ [51, 52]. This is why PEI retains buffering capacity in the endosomal pH range despite having nominal pKₐ values above 7.4: the crowding effect creates a broad protonation profile rather than a sharp transition, allowing continued proton absorption as pH drops from 7.4 to 5.0. These polymers bind RNA tightly at all physiologically relevant pH values, making them effective at condensation and protection but poor at cargo release regardless of whether escape occurs.

Polymers engineered with lower pKₐ values offer more selective endosomal protonation. Poly(β-amino esters) (PBAEs), with reported pKₐ values between 6 and 7, gain substantial charge only within the endosomal pH range, which accounts for their lower cytotoxicity relative to PEI: they do not strongly disrupt the plasma membrane at physiological pH [53]. The same principle of pKa-tuned endosomal activation applies to ionizable lipids in LNP formulations [12, 14]. Poly(2-(diethylamino)ethyl methacrylate) (PDEAEMA, pKa ~ 7.0) and poly(2-(diisopropylamino)ethyl methacrylate) (PDPAEMA, pKa ~ 6.3) follow a similar pattern [54]. However, the fundamental tradeoff remains: at any pH where these polymers have acquired sufficient charge to destabilize endosomal membranes, they have also acquired sufficient charge to complex RNA. The pKₐ can be tuned to shift the onset of escape, but it cannot decouple escape from binding because both are driven by the same protonation events. Breaking this coupling requires chemistries that introduce a second trigger or a temporal delay between escape activation and charge neutralization, the strategies reviewed in Sects. "pH-responsive and charge-shifting polymers as escape–release coupling devices" and "Non-pH Stimulus-Responsive Strategies: Do Alternative Triggers Resolve the Tradeoff?". Table 1 maps the key design parameters that govern this conflict, showing how each favors either escape or release but not both.

**Table 1:** Comparison of Escape-Favoring *vs.* Release-Favoring Design Features in Polymeric Carriers

Design Parameter	Favors Endosomal Escape	Favors Cargo Release	Tradeoff Consequence
Charge density	High—strong membrane disruption	Low/neutral—weak electrostatic grip	Cannot maximize both simultaneously from same ionizable groups
pKa of ionizable groups	> 7—charge present at physiological and endosomal pH	6–6.5—selective endosomal protonation, but still tightens binding at escape-competent pH	Shifting pKa moves the onset but does not decouple escape from binding
Polymer degradability	Non-degradable—maintains structural integrity during trafficking	Degradable—enables timed carrier disassembly	Degradation must not occur before escape is complete
Hydrophobicity	High—promotes membrane insertion and destabilization	Low—reduces nonspecific membrane interaction, favors hydration and release	Hydrophobic polymers may enhance escape but retard aqueous-phase dissociation
N/P ratio	High—excess charge for uptake and escape	Low—fewer electrostatic contacts, easier release	Optimum is system-specific, shifts with cargo size and cell type
PEGylation	Low—preserves charge-mediated membrane interaction	High—reduces nonspecific binding, may weaken RNA complexation	PEG inhibits escape while potentially aiding release—another coupled tradeoff
Stimulus-responsive degradation	Absent—stable carrier throughout	Present—timed disassembly triggered by pH, redox, or enzyme	Must be tuned so degradation occurs after escape but before lysosomal commitment

#### The N/P Ratio Dilemma Reflects the Tradeoff Quantitatively

The nitrogen-to-phosphorus (N/P) ratio, the molar ratio of ionizable amines on the polymer to phosphate groups on the nucleic acid, is the primary formulation lever for balancing complexation and delivery. The conflicting reports in the literature on optimal N/P ratios are themselves evidence of the tradeoff.

The conflict is systematic rather than random. For 25 kDa branched PEI delivering plasmid DNA, transfection efficiency in HeLa cells peaks around N/P 10 but drops at higher ratios where cytotoxicity from excess free polymer offsets gains in escape [37]. Yet for the same polymer in primary neurons or other hard-to-transfect cells, higher N/P ratios are required simply to achieve sufficient uptake and escape, despite the associated toxicity and reduced release [55]. PBAEs present a different pattern: because their lower charge density provides weaker RNA condensation per unit mass, effective siRNA delivery often requires N/P ratios of 40 or higher, whereas mRNA delivery can succeed at N/P 10–20. This difference is attributable to the greater number of electrostatic contacts that the longer mRNA chain makes with the polymer, which provides adequate condensation at lower charge excess [42, 56]. PEGylation adds another layer. PEG shields reduce the effective charge available for membrane interaction, meaning PEGylated systems often require higher N/P ratios to achieve the same level of escape as unPEGylated counterparts, but the additional polymer further inhibits release [2]. The role of excess polymer at high N/P ratios deserves additional consideration. Several studies have shown that at N/P ratios above charge neutralization, a substantial fraction of the polymer exists as free, uncomplexed chains rather than as part of the polyplex structure [57, 58]. These free polymer chains are not inert bystanders. Evidence suggests they contribute independently to endosomal escape by destabilizing endosomal membranes without being bound to the nucleic acid cargo [59]. This complicates the interpretation of N/P ratio optimization studies, because the observed improvements in transfection at high N/P ratios may reflect free polymer-mediated escape rather than improved polyplex function. It also raises a translational concern: free cationic polymer that remains in close proximity to the polyplex in a culture dish may disperse rapidly upon systemic administration, diluting the escape-enhancing effect that was observed *in vitro*. Whether the free polymer fraction that drives escape in cell culture remains associated with the polyplex long enough to reach the target endosome *in vivo* has not been rigorously tested.

These observations show that the "optimal" N/P ratio is not a fixed property of a polymer but a system-specific compromise between escape and release that shifts with polymer chemistry, cargo size, cell type, and formulation excipients. The conflicting literature does not reflect poor experimental design. It reflects the genuine impossibility of simultaneously maximizing both escape and release through charge titration alone. However, the relationship between binding strength and bioavailability is not always monotonic. Parsons *et al.* showed that stronger electrostatic binding in block ionomer complexes actually improved siRNA delivery to RISC, likely because tighter complexation protected siRNA from cytosolic RNA-binding proteins and ribonucleases that would otherwise strip loosely bound cargo before RISC loading could occur [60]. This suggests that for small cargo like siRNA, the risk of cytosolic degradation after release can outweigh the risk of kinetic trapping, and the optimal binding strength depends on the competition between RISC loading and non-specific RNA-binding protein association.

#### Most Escaped RNA Remains Carrier-bound in the Cytosol

The most consequential and least appreciated aspect of the tradeoff is that cytosolic delivery, as typically measured, overstates functional delivery. Nucleic acid cargo frequently remains associated with its polymeric carrier even after successful endosomal escape.

Kim *et al.* systematically investigated this using mixed polycation systems of α-poly(L-lysine) and ε-poly(L-lysine), varying complexation tightness from loose to tight [61]. Tighter polyplexes delivered more pDNA into cells and into the nucleus than looser polyplexes, but produced *worse* gene expression because the DNA could not be released from the carrier for transcription. The study further showed that larger polyplexes exhibited slower cytosolic transport, linking polyplex size to both trafficking kinetics and release efficiency. This work directly demonstrates that complexation strength and delivery efficiency are not monotonically related. There is an optimum beyond which tighter binding actively suppresses the biological function of delivered cargo.

For bioreducible polymers, Hwang, Kang, and Bae showed that polyplex decomplexation rate, measured by dithiothreitol (DTT)- and heparin-induced release of YOYO-1-labeled pDNA, varied with the content of reducible disulfide bonds, and that transfection efficiency tracked with decomplexation rate rather than with cellular uptake, cytotoxicity, or particle size [35]. Polymers with intermediate decomplexation kinetics (fast enough to release cargo but slow enough to protect it during trafficking) outperformed both rapidly and slowly dissociating variants. Nuclear localization of delivered pDNA showed a similar optimum, confirming that the decomplexation–transfection correlation held across the entire intracellular delivery pathway.

The problem is compounded for larger nucleic acid cargo. Maugeri and colleagues reported that the cytosolic concentration of mRNA following LNP-mediated delivery is approximately half that of siRNA from the same system [62]. Although this observation was made in LNP rather than polyplex systems, the underlying physics (more electrostatic contacts along longer polyanionic chains, greater steric entanglement, and increased difficulty threading large molecules through transient membrane pores) applies with even greater force to polyplexes, where charge densities are higher and polymer–RNA contacts more numerous than lipid–RNA contacts [42]. This cargo-size dependence is analyzed in detail in Sect. "Cargo-Specific Considerations: Why One Size Does Not Fit All".

The functional consequence is clear: free RNA in the cytosol is the only form capable of engaging the RISC (for siRNA) or ribosomes (for mRNA). RNA that remains polymer-bound after escape is functionally inert. It has survived the endosome but cannot execute its therapeutic program. The ~ 1–2% cytosolic delivery efficiency commonly cited for nucleic acid nanocarriers likely overstates functional delivery [63, 64], because a substantial fraction of the RNA that reaches the cytosol may still be complexed. The true "functional delivery efficiency," the fraction of administered RNA that reaches the cytosol in a free, biologically active form, is almost certainly lower. The field currently lacks standardized assays to measure it (Sect. "Measuring Escape and Release: The Assay Gap").

Together, these observations establish that endosomal escape and nucleic acid release are not sequential, independent steps but are coupled through the same electrostatic and pH-dependent interactions. Optimizing for one at the expense of the other yields systems that are either potent escapers that trap their cargo, or efficient releasers that never leave the endosome. The remainder of this review examines polymer design strategies that attempt to resolve this tension through cargo-matched design (Sect. "Cargo-Specific Considerations: Why One Size Does Not Fit All"), stimulus-responsive chemistries that temporally decouple escape from release (Sects. "pH-responsive and charge-shifting polymers as escape–release coupling devices" and "Non-pH Stimulus-Responsive Strategies: Do Alternative Triggers Resolve the Tradeoff?"), architectural and formulation approaches (Sect. "Polymer Architecture and Formulation: Structural Approaches to Decoupling Escape from Release"), and improved assays for measuring both processes simultaneously (Sect. "Measuring Escape and Release: The Assay Gap").

### Application-Specific Targets: How Much Functional Delivery Is Enough?

The functional delivery efficiency required for therapeutic success is not a fixed target. It varies substantially by application in ways that reshape where the escape–release balance point should sit. Gene silencing with siRNA is catalytic: once loaded into RISC, a single siRNA molecule can directly cleave many transcripts, and comparatively modest cytosolic copy numbers are often sufficient to produce durable knockdown [65]. For CRISPR-based gene editing delivered as ribonucleoprotein or as Cas9 mRNA, only a transient pulse of nuclease activity is required, and prolonged expression is often actively undesirable because off-target editing accumulates over time [66, 67]. Gene replacement and protein-expression therapies using mRNA or plasmid DNA impose the opposite requirement: sustained, high-level protein production must be achieved and maintained across many cells, so both the magnitude and duration of functional delivery matter [68, 69]. Nucleic acid vaccines sit in between, needing an antigen-expression pulse high enough to prime an immune response but time-limited by intrinsic mRNA turnover [70].

This range of requirements has direct implications for carrier design. The same “1–2% cytosolic delivery” figure translates into therapeutic success or failure very differently across cargo types and clinical goals. A carrier optimized for rapid, complete cargo release may serve siRNA well but under-deliver for mRNA expression, where sustained protein output can benefit from a slow-release depot effect (Sect. "Cargo-Specific Considerations: Why One Size Does Not Fit All"). Systems designed to maximize peak cytosolic exposure may be well suited to editing but poorly matched to therapies requiring durable expression. The escape–release balance point is therefore not merely cargo-dependent but application-dependent: what "enough" functional delivery looks like must be defined for the specific therapeutic goal before a design can be judged.

## Cargo-Specific Considerations: Why One Size Does Not Fit All

The escape–release tradeoff described in Sect. "The Mechanistic Basis of the Escape–Release Tradeoff" is not a fixed conflict. Its severity and the position of the optimum shift depend on the physical and biological properties of the nucleic acid cargo. Small, rigid, double-stranded siRNA (~ 14 kDa, 42 phosphate groups) presents a very different binding surface to a polymeric carrier than large, flexible, single-stranded mRNA (300–1500 kDa, thousands of phosphate groups) or massive supercoiled plasmid DNA [71, 72]. CRISPR-Cas ribonucleoproteins (RNPs), which combine a protein and a guide RNA into a single complex (~ 150–200 kDa), introduce additional constraints related to conformational integrity [73]. Because each cargo type makes a different number of electrostatic contacts with the polymer, occupies a different volume within the polyplex, and must reach a different intracellular destination, the same carrier chemistry will produce a different escape–release balance for each payload.

### Cargo Size Determines Binding Cooperativity and the Risk of Kinetic Trapping

**Fig. 2:**
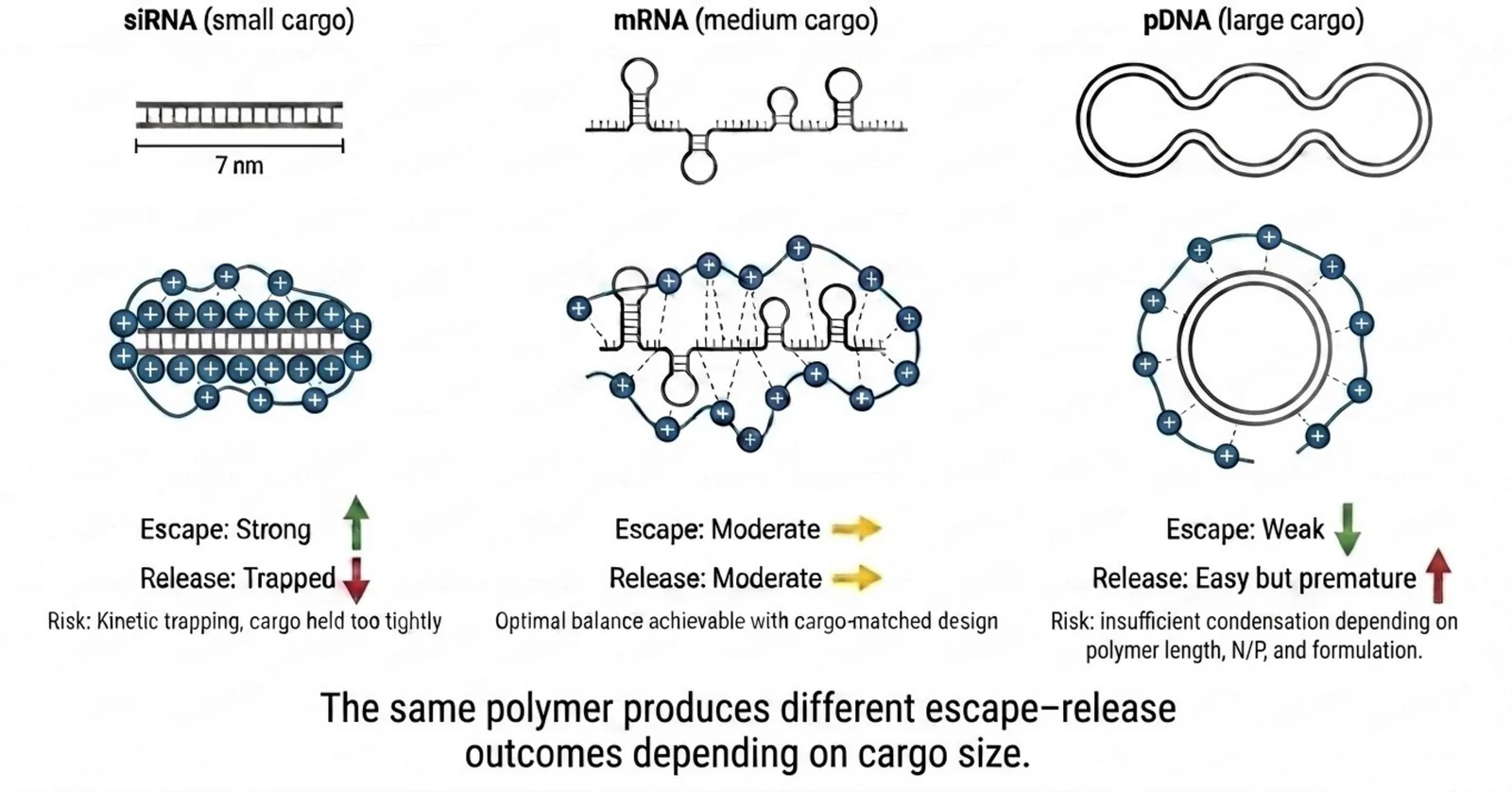
The same polymer produces different escape–release outcomes for different cargo sizes. When a single cationic polymer is applied across cargo types, it over-condenses small siRNA (leading to kinetic trapping and difficult release), achieves a moderate balance with mRNA. For pDNA, whether condensation is adequate depends on polymer length, N/P ratio, topology, and formulation, so the outcome is context-dependent rather than fixed by cargo size alone. No single polymer design simultaneously optimizes both escape and release across all cargo types.

The relationship between nucleic acid size and polymer valency directly governs how tightly a polyplex holds its cargo. For large nucleic acids such as pDNA, the high density of negative charge distributed over thousands of bases requires strong, cooperative electrostatic binding to achieve condensation into nanoscale polyplexes [74, 75]. However, the same cooperative binding that enables condensation of large cargo can trap smaller nucleic acids. For siRNA, with only 42 phosphate groups, a high-valency polymer provides far more cationic contacts than needed for condensation, creating kinetic trapping: the cargo is held so tightly that it cannot dissociate for RISC loading after escape [36, 76]. Lower-valency carriers provide weaker but more dynamic binding, facilitating release of small cargo while still offering adequate protection during trafficking [36]. The tradeoff optimum therefore shifts with cargo size. Large cargo tolerates and requires tighter binding, while small cargo demands looser complexation that may come at the cost of reduced escape efficiency. Figure 2 illustrates this relationship, showing how the same polymer produces different escape–release outcomes depending on cargo size.

### Optimal Polymer Parameters are Cargo-specific, Not Universal

The most direct evidence for cargo-dependent optimization comes from a comprehensive library screen of PEI-based copolymers by Blakney *et al.*, who tested polymer chain length and charge density across multiple nucleic acid types [77]. For pDNA, the highest transfection efficiency was obtained with a long, highly charged copolymer (83 kDa, 100% charge density). For mRNA, a smaller polymer with reduced charge performed best (45 kDa, 80% charge density). Self-amplifying replicon RNA (RepRNA) required an intermediate configuration (72 kDa, 100% charge density), and its design space was significantly narrower than that for either pDNA or mRNA [77]. These results confirm that the escape–release balance point is different for each cargo: the polymer that maximizes pDNA delivery over-condenses mRNA, and the polymer that releases mRNA efficiently cannot adequately protect RepRNA. Treating any single set of polymer parameters as universally "optimal" ignores this cargo dependence.

### Cargo Topology and Secondary Structure Affect the Tradeoff Independently of Size

Beyond molecular weight, the physical conformation of the nucleic acid influences both polyplex architecture and intracellular processing. Non-viral carriers can encapsulate both supercoiled and linearized plasmid DNA into nanoparticles of comparable size, but supercoiled DNA produces substantially higher transgene expression [75]. This difference does not arise from delivery efficiency. Rather, linearized DNA with exposed 5'-phosphate ends resembles damaged DNA and triggers inhibitory cellular responses [75]. Minicircle DNA, which lacks the bacterial backbone of conventional plasmids, shows enhanced intracellular diffusion and nuclear entry, leading to more efficient and sustained expression [18]. These observations show that the cargo's physical form can shift the bottleneck from escape or release to downstream processing steps that lie outside the carrier's control.

Gilbert et al. demonstrated a related effect at the level of polyplex formation itself: the extent of intramolecular base pairing in a nucleic acid cargo significantly affects the internal architecture and colloidal stability of the resulting nanoparticle [78]. Highly base-paired cargoes induced spontaneous polyplex aggregation that less structured cargoes did not, suggesting that cargo secondary structure can alter the balance between condensation stability and release propensity before the polyplex ever encounters an endosome [78].

### How Each Cargo Type Shifts the Tradeoff

The general principles above play out differently for each major cargo class, and recognizing these differences is essential for interpreting the stimulus-responsive strategies reviewed in Sects. "pH-responsive and charge-shifting polymers as escape–release coupling devices" and "Non-pH Stimulus-Responsive Strategies: Do Alternative Triggers Resolve the Tradeoff?".

For mRNA, the tradeoff is dominated by a narrow release window. mRNA acts in the cytoplasm, bypassing the need for nuclear entry, but it is highly susceptible to degradation by cytoplasmic ribonucleases [79, 80]. Its large size creates extensive electrostatic contacts with the polymer, requiring relatively tight condensation for protection during trafficking. Yet delayed release after escape leads to mRNA degradation before translation can begin [80, 81]. The escape–release balance for mRNA therefore favors carriers that release cargo rapidly upon cytosolic entry, even at the cost of reduced extracellular stability. Acid-responsive polymers formulated with lipids have demonstrated this principle, with endosomal RNA dissociation increasing transfection up to 5.4-fold compared to conventional LNPs [82]. Nucleoside modifications such as N1-methylpseudouridine are now standard in clinical mRNA formulations, but their primary contributions are to reduce innate immune activation and to enhance translation efficiency, not to provide dramatic nuclease resistance [70]. The rapid-release window argument is therefore only modestly relaxed for modified mRNA, which remains larger and more electrostatically encumbered than siRNA and continues to depend on carriers that release cargo efficiently once in the cytosol.

For siRNA, the tradeoff centers on partial decomplexation. These small, double-stranded molecules (21–23 nucleotides) act through RISC in the cytoplasm [83]. Over-stable complexes prevent siRNA from loading into RISC, while excessive decomplexation during trafficking exposes the cargo to degradation. Because siRNA makes relatively few electrostatic contacts with the polymer compared to mRNA or pDNA, the risk of kinetic trapping is high when carriers designed for larger cargo are repurposed for siRNA delivery. The tradeoff optimum for siRNA lies closer to the "release-favoring" end of the spectrum than for any other cargo type, favoring carriers with moderate binding strength and built-in degradation mechanisms such as the bioreducible systems discussed in Sect. "Cytosol-Triggered Release: Do Bioreducible Carriers Resolve the Tradeoff?". However, clinically used siRNAs incorporate chemical modifications (2'-O-methyl, 2'-fluoro, phosphorothioate backbones) that substantially increase nuclease resistance [84–86]. For these modified sequences, pre-RISC degradation in the cytosol is unlikely to be a major loss pathway, and the release window after escape can be considerably wider than for unmodified siRNA, relaxing the kinetic trapping constraint. Modified siRNAs can also accumulate in endo-lysosomal depots and release slowly over time into the cytosol, producing durable knockdown from a small fraction of the internalized dose [11, 14, 87]. This depot behavior means that carriers with slower or partial release kinetics may still deliver therapeutically useful siRNA activity, and it suggests that maximal cytosolic exposure at short times is not the only viable design target for siRNA.

For CRISPR-Cas RNPs, the tradeoff is complicated by the need to preserve protein conformation. RNPs require both guide RNA and Cas protein integrity for activity, and both components are sensitive to acidic pH and high ionic strength [73]. Carriers that achieve escape through aggressive membrane destabilization or strong proton buffering may denature the Cas protein in the process. RNP delivery therefore demands ultra-mild release mechanisms, often mediated by redox or enzymatic triggers, that protect the complex from the harsh endosomal environment while still enabling efficient cytosolic release without conformational damage [73]. This places RNP delivery at the most constrained point on the escape–release spectrum, where both excessive escape activity and excessive binding are harmful.

### Nuclear Entry Adds a Third Variable for DNA Payloads

For siRNA and mRNA, the therapeutic site of action is the cytosol, and successful escape followed by release completes the delivery task. For pDNA, a third barrier remains: the cargo must cross the nuclear envelope. Because pDNA far exceeds the ~ 50 kDa diffusion limit of the nuclear pore, nuclear entry depends on active transport mechanisms [18]. Three distinct routes have been described. In dividing cells, the nuclear envelope disassembles during mitosis and reforms around cytoplasmic contents, providing a passive route for pDNA entry that is not available in non-dividing cells such as neurons, hepatocytes, or terminally differentiated immune cells [88]. For non-dividing cells, active nuclear import must be engineered either into the cargo or into the carrier. DNA nuclear targeting sequences (DTS) are short cis-acting sequences that recruit endogenous transcription factors and importins to the plasmid backbone, exploiting the cellular nuclear import machinery to shuttle DNA into the nucleus [89]. Nuclear localization signal (NLS) peptides can be conjugated to the polymeric carrier or covalently attached to the plasmid to achieve a similar effect through direct importin engagement [90]. These approaches show that nuclear entry can be engineered as a property of the cargo (through DTS) or of the carrier (through NLS conjugation), and it is at least partially separable from the escape–release tradeoff itself.

The tradeoff nevertheless compounds for pDNA in a way it does not for RNA payloads. pDNA requires sufficiently tight condensation to protect against cytosolic nucleases and to enable passage through nuclear pore complexes or association with mitotic chromatin, yet the same tight condensation resists the decomplexation required for transcription. Whether the balance shifts toward retention (tight polyplexes that reach the nucleus at the cost of transcriptional accessibility) or toward release (efficient decomplexation at the cost of nuclear delivery) depends on polymer length, N/P ratio, topology, and formulation. pDNA is therefore the most constrained cargo in the escape–release framework, layering a third sequential barrier onto the two that already define the tradeoff for RNA payloads.

Beyond carrier and cargo, endocytic route is a third variable that shifts where the escape–release balance point should sit. Nanoparticles can enter cells through clathrin-mediated endocytosis, caveolae, macropinocytosis, or clathrin- and caveolae-independent pathways, and the route of uptake determines the kinetics of endosomal maturation, the pH profile encountered by the cargo, and the ultimate trafficking fate of the vesicle [91]. Clathrin-mediated endocytosis funnels particles into rapidly acidifying early endosomes and then to late endosomes and lysosomes, imposing a short window in which pH-triggered escape must occur. Caveolar routes are typically slower to acidify and can bypass canonical lysosomal degradation, offering a longer window but a weaker pH signal. Macropinocytosis produces large, leaky endosomes with distinct trafficking dynamics. Because targeting ligands and surface chemistry can bias uptake toward one route or another, the same polyplex can experience very different intracellular environments depending on the receptor engaged and the cell type encountered [91]. Optimal escape–release design is therefore route-dependent as well as cargo-dependent, and reports of the "best" trigger pKa or release kinetic for a given polymer class often reflect the specific endocytic pathway probed by the assay rather than a universal design principle.

These cargo-specific considerations reinforce the argument that the escape–release tradeoff cannot be addressed with a single polymer design. The strategies reviewed in Sects. "pH-responsive and charge-shifting polymers as escape–release coupling devices" and "Non-pH Stimulus-Responsive Strategies: Do Alternative Triggers Resolve the Tradeoff?", which attempt to temporally decouple escape from release through pH-responsive or orthogonal trigger chemistries, must be evaluated against the specific binding, release, and downstream processing requirements of the intended payload.

## pH-responsive and Charge-shifting Polymers as Escape–release Coupling Devices

In this section, we will consider pH-responsive polymers as carriers that become more protonated and membrane-active as endosomal pH falls, whereas we consider charge-shifting polymers as carriers that weaken carrier-cargo association after entry by converting from cationic to neutral or anionic states. Although the literature often groups both under broad “charge-shifting” design language, they are intended to solve different bottlenecks. pH-responsive systems primarily control where and when membrane activity begins, while charge-shifting systems primarily control where and when electrostatic binding is relaxed. Their importance therefore lies not simply in whether they enhance endosomal escape, but in how pH-responsive systems regulate membrane activation and how charge-shifting systems regulate intracellular cargo release, with some carrier designs combining both functions according to the needs of the payload [4, 5, 17, 22, 92].

Polymer studies using dual-color fluorescence fluctuation spectroscopy and FRET-based unpacking assays have confirmed that release is a measurable intracellular variable, not merely an inference from final transfection outcomes (Sect. "Measuring Escape and Release: The Assay Gap") [26]. QD-FRET studies further showed that the onset of intracellular unpacking can be directly detected in living cells, making release a measurable mechanistic variable rather than something inferred only from final transfection [27, 28]. In PEI-PEG-TAT polyplexes, transfection efficiency correlated positively with uptake rate but negatively with the rate of unpacking in endo/lysosomal compartments, showing that release in the wrong compartment can be actively detrimental [4]. These polymer-specific results are important because they show that the central issue is not simply uptake or even endosomal damage, but how uptake, escape, unpacking, and downstream processing are kinetically coupled [26–28, 93].

### pH-responsive Polymers: Tuning the Activation Zone of Membrane Activity

pH-responsive polymers exploit the drop in pH that accompanies endosomal maturation. As the carrier encounters progressively more acidic compartments, protonation can alter its solubility, conformation, hydrophobicity, or membrane interaction. In practice, this means that acidification can drive several different processes: exposure of membrane-active groups, particle disassembly, PEG shedding, or redistribution of polymer within the complex [17, 22, 32]. The important question is therefore not whether a carrier is “pH-responsive” in the abstract, but where along the endocytic pathway that response is triggered and what the response actually accomplishes.

This is why pKa should be treated cautiously. Effective carrier pKa does not act as a precise clock, but it does bias the activation zone of the carrier. Higher effective pKa can favor protonation and membrane activity in earlier endosomal compartments, whereas lower effective pKa can delay activation into later, more acidic compartments [12, 17, 22]. For cytosolic cargos such as siRNA and mRNA, early-to-maturing endosomal activation is often attractive because it may reduce exposure to deeper degradative routing and avoid some of the biological costs associated with later lysosomal rupture. However, this should not be treated as a universal law. Earlier activation may also increase premature membrane activity, nonspecific interactions, tighter residual binding, and toxicity [17, 22]. Thus, pKa is better understood as an activation-zone bias than as a universal measure of carrier quality.

The pHlexi nanoparticle series illustrates both the promise and the complexity of this strategy. By tuning the relative contributions of PDEAEMA and PDPAEMA, the authors generated particles with different disassembly pH values and observed corresponding changes in escape-like behavior [32]. Importantly, the relationship was not monotonic: strong calcein leakage occurred at both high and low disassembly pH, whereas intermediate formulations were less effective [32]. This result argues against a simplistic “earlier is always better” interpretation. Instead, it suggests that varying activation thresholds may shift not only *when* a carrier becomes active, but also *how*it perturbs endosomal membranes. The same study also provides a key caution: the highest-pH disassembling PDEAEMA-rich particles were the most toxic, showing that aggressive early activation can carry a clear biological cost [32].

The SLEEQ assay (split luciferase endosomal escape quantification) sharpens this point by directly quantifying cytosolic access rather than relying on small-molecule leakage. In that study, pH-responsive nanoparticles disassembling around pH 6.4 showed substantially greater cytosolic delivery than particles disassembling around pH 5.6, even though both produced endosomal leakage in indirect assays [30]. This is important because it shows that membrane leakage and productive cytosolic access are not interchangeable. It also supports a staged design logic in which one carrier regime supports extracellular condensation and persistence, while another regime biases endosomal membrane interaction toward a more productive window [17, 30].

Another important point is that acidification may help one bottleneck while worsening the next. Several reviews emphasize that pH-responsive carriers may escape through a mixture of buffering-associated osmotic stress, direct membrane interaction, or particle disassembly into many subunits [17, 22, 38]. Polymer studies also suggest that acidification can promote polymer shedding or structural rearrangements that increase membrane activity while simultaneously changing how tightly the remaining complex holds the nucleic acid [17]. Thus, even when acidification improves endosomal membrane damage, it does not automatically guarantee productive downstream release. A pH-responsive carrier can still fail if it destabilizes endosomes efficiently but retains the cargo too tightly afterward [17, 22].

A polymer's pKa provides one route to break this coupling without requiring a separate release chemistry. For carriers with effective pKa in the endosomal range, approximately 6 to 7 as seen in PBAEs, PDEAEMA, and PDPAEMA, the polymer is substantially cationic in the acidic endosome but only partially protonated at cytosolic pH 7.4. Endosomal escape therefore exposes the carrier to a pH environment that reverses the same protonation event driving both membrane activity and cargo binding, deprotonating the amines and weakening the electrostatic grip on the nucleic acid [56]. In this case the escape event itself functions as the release trigger, and no orthogonal chemistry (redox, ROS, or enzymatic) is required to break the coupling. This is one of the more promising expressions of escape-coupled release available with a single-component polymer, and it argues that the choice of effective pKa should be treated as a release-engineering decision as much as an escape-engineering one. The effect is only as strong as the pKa is well-placed, however: polymers with pKa well above 7.4, such as PEI and poly(L-lysine), remain heavily cationic even at cytosolic pH and derive little release benefit from cytosolic exposure, which is why they depend on the separate charge-shifting or degradation strategies discussed in Sect. "Charge-shifting polymers: programming release after entry".

As discussed in Sect. "The Mechanistic Basis of the Escape–Release Tradeoff", the proton sponge hypothesis is historically important but likely incomplete. The more useful framework is to ask how pH changes alter the sequence of carrier state changes and whether those changes support productive delivery for the specific payload [22, 31].

### Charge-shifting Polymers: Programming Release After Entry

**Fig. 3:**
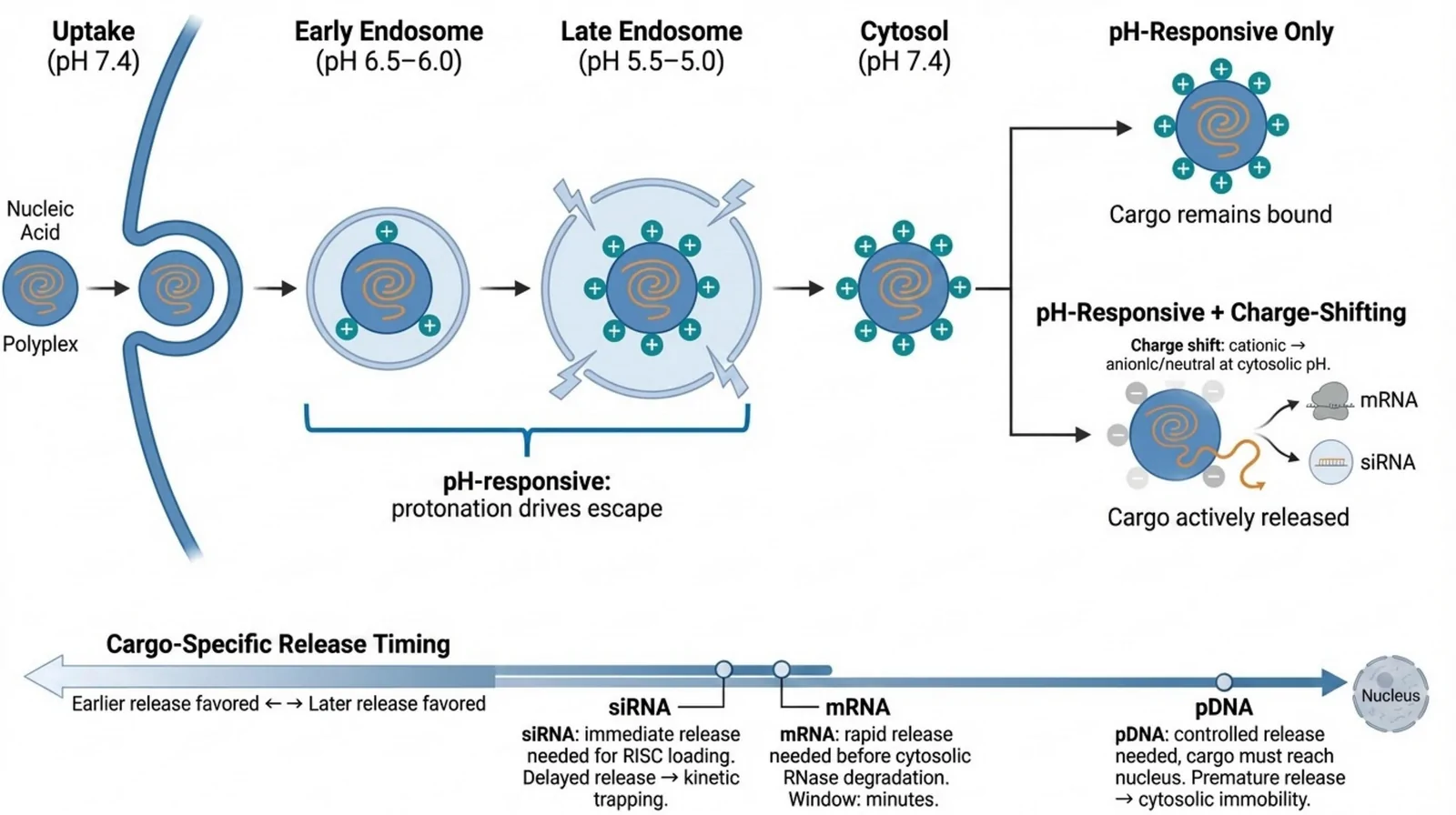
pH-responsive and charge-shifting polymeric carriers share the same endosomal trafficking pathway but diverge after escape. Both strategies rely on amine protonation during endosomal acidification to drive membrane destabilization. After escape, pH-responsive-only carriers retain cationic charge and hold cargo through persistent electrostatic binding. Charge-shifting carriers undergo an additional chemical transition at cytosolic pH, converting from cationic to anionic or neutral and actively expelling cargo. The bottom panel shows cargo-specific release timing: siRNA requires immediate release for RISC loading, mRNA requires rapid release before RNase degradation, and pDNA requires controlled release to maintain protection during nuclear transit.

If pH-responsive polymers primarily tune membrane activity, charge-shifting polymers primarily tune carrier-cargo dissociation after entry. These systems are especially attractive because they directly address one of the major liabilities of conventional cationic carriers: the same positive charge that promotes condensation and uptake can later hinder intracellular release and contribute to toxicity [5, 33]. Rather than relying mainly on acidification to trigger membrane activity, charge-shifting systems are designed to weaken electrostatic binding after entry by hydrolysis, rearrangement, or cleavage. In that sense, they solve the binding problem more directly than the activation problem. Figure 3 illustrates this distinction, showing how both strategies share the same endosomal trafficking pathway but diverge at the point of cytosolic entry.

The clearest example is provided by charge-altering releasable transporters (CARTs). The original CART study argued that delivery systems adapted from DNA or siRNA work can fail for mRNA not necessarily because uptake is poor, but because intracellular release is inadequate for productive translation [33]. CARTs address this by functioning initially as cationic carriers that complex and protect mRNA, then undergoing a charge-neutralizing rearrangement to produce neutral products and facilitate release [33]. Crucially, uptake alone did not explain performance: nonimmolative controls showed similar uptake but little or no protein expression. The decisive variable was whether the carrier transitioned from a protective cationic state to a release-permissive state after entry [33]. That result strongly supports the idea that productive delivery depends on intracellular release kinetics, not uptake alone.

Subsequent work has broadened this principle. Ser-CARTs demonstrated that the charge-altering concept is not restricted to a single scaffold but can be implemented in other degradable backbones that likewise support efficient mRNA delivery *in vitro* and *in vivo* [94]. Lipid-modified CARTs extended the same logic to pDNA, showing that charge-altering systems can support functional DNA delivery and stable transfection [95]. More recent β-amido-carbonate CART variants further show that structurally tuned charge-altering systems can enhance mRNA delivery to difficult targets such as primary T cells while also altering *in vivo* tropism [96]. Collectively, these studies make it clear that charge-shifting is not just an isolated chemical trick, but a broader design class built around programmed weakening of electrostatic binding after entry [33, 94–96].

The broader charge-conversion literature supports this view. Dutta *et al.* note that persistent cationic charge aids condensation and escape, but can also drive toxicity and prevent intracellular release [2]. However, reducing cationic character is not automatically beneficial. Weakening charge improves release and safety but may also reduce membrane activity unless a separate escape mechanism is included [2]. This is why pH-responsive and charge-shifting systems should not be treated as interchangeable. A pH-responsive carrier can fail if it escapes but never releases its cargo. A charge-shifting carrier can fail if it releases cargo but cannot escape. The most effective systems may need both functions, but the two should be designed and evaluated separately [2, 17, 33].

A second reason charge-shifting systems matter is safety. Classical PEI toxicity is increasingly understood as temporally multipart, with an early membrane-associated component and a later intracellular component involving mitochondria, oxidative stress, and apoptosis [5, 97–99]. This raises the possibility that persistent intracellular cationicity is itself part of the safety problem. In this context, degradable, bioreducible, or charge-converting systems may improve biocompatibility not only by promoting release, but by shortening the lifetime of strongly cationic intracellular material [2, 97–99]. Charge-shifting systems are therefore attractive both as release-promoting materials and also as materials that may reduce the duration of intracellular cationic exposure [2, 97–99].

### Responsive Carrier Design Should Follow the Bottleneck

Viewed through this lens, pH-responsive and charge-shifting polymers should not be treated as a menu of equivalent “smart carriers.” They address different bottlenecks and should be judged accordingly. pH-responsive systems are most useful when the main challenge is activating membrane interaction in a productive endosomal compartment. Charge-shifting systems are most useful when the main challenge is weakening carrier-cargo association after entry so that escape is followed by release rather than continued sequestration.

This is why the assay framework discussed in Sect. "Measuring Escape and Release: The Assay Gap" is a direct consequence of the argument made here: if responsive polymers are solving different timed steps, the field needs measurements that can distinguish those steps rather than collapsing them into a single delivery readout [22, 25–28, 30, 93].

The most useful design rules emerging from current work are therefore straightforward even if difficult to implement. First, optimize for productive function rather than maximal escape in the abstract. Second, treat effective carrier pKa as an activation-zone bias, not a universal score. Third, match escape and release kinetics to the payload’s downstream bottleneck rather than assuming one profile will suit all nucleic acids [2, 4, 5, 11, 17, 22, 33, 92].

## Non-pH Stimulus-Responsive Strategies: Do Alternative Triggers Resolve the Tradeoff?

Sect. "pH-responsive and charge-shifting polymers as escape–release coupling devices" examined pH-responsive strategies (charge-shifting polymers, pKₐ-tuned systems, and charge-altering releasable transporters) that attempt to temporally decouple endosomal escape from nucleic acid release using the same pH gradient that creates the problem. A natural question follows: can stimuli other than pH break the coupling more cleanly? Reduction-sensitive disulfide cleavage, oxidation-triggered degradation, and enzyme-responsive linkages all operate through chemical mechanisms orthogonal to the protonation events that drive escape, making them attractive candidates for selective cargo release without compromising membrane destabilization. However, the therapeutic value of any stimulus-responsive release strategy depends not only on the chemistry of the trigger but on where and when that trigger is available relative to the escape event. This spatial and temporal alignment problem, not yet extensively addressed in the stimulus-responsive delivery literature, determines whether a given strategy genuinely resolves the escape–release tradeoff or merely relocates the bottleneck.

### The Spatial Mismatch Problem

The intracellular environment is not a uniform solution. Redox potential, enzyme activity, and reactive oxygen species concentrations are compartmentalized, with sharp gradients across organellar membranes that create fundamentally different chemical environments within the compartments of a single cell [100]. For stimulus-responsive polymeric carriers, this compartmentalization imposes a spatial constraint: the trigger that degrades the carrier must be present in the same compartment as the polyplex at the moment when release would be productive, that is, after escape has occurred but before the RNA is degraded or sequestered.

**Fig. 4:**
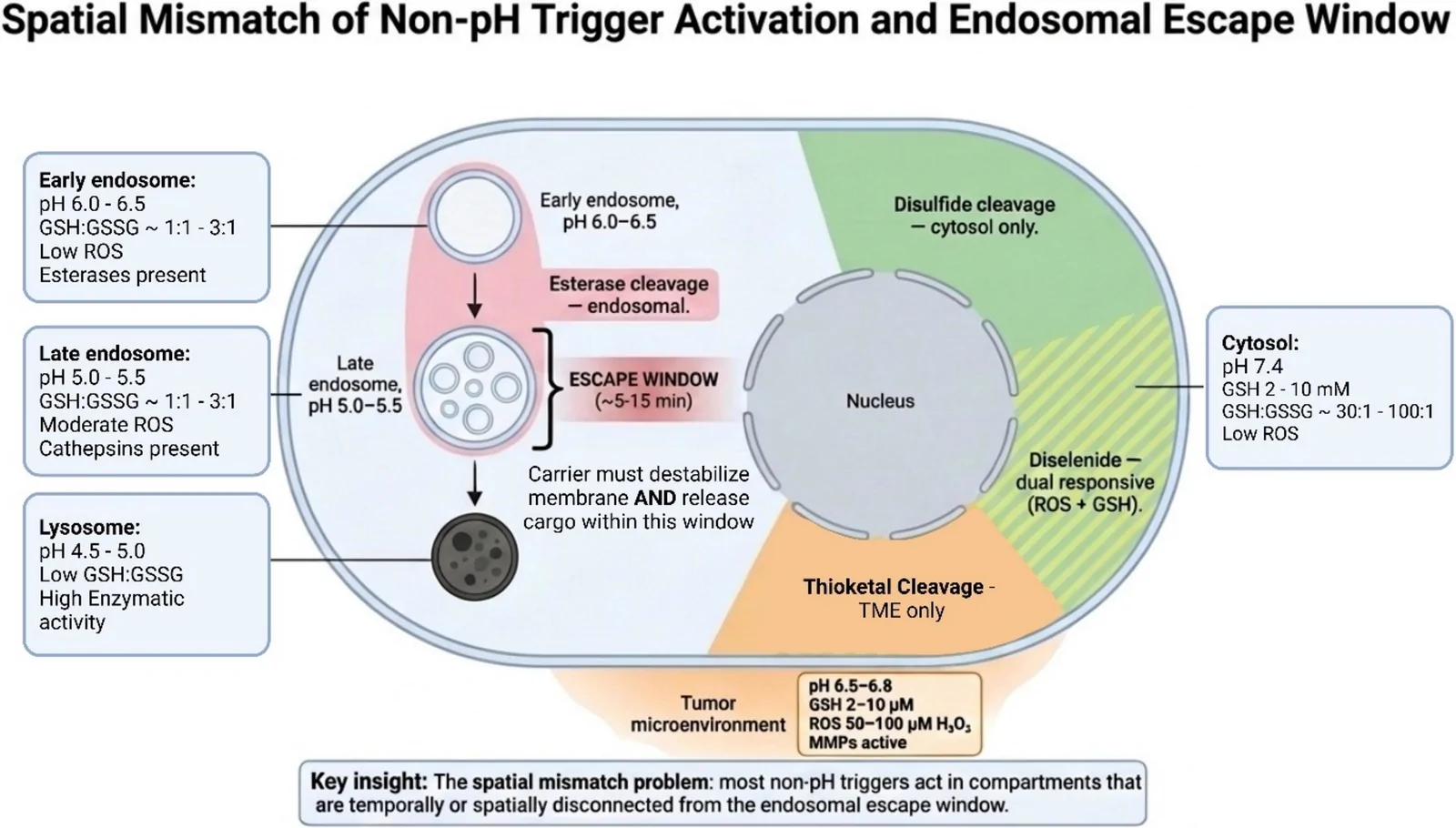
Spatial mismatch between non-pH trigger activation sites and the endosomal escape window. The chemical environment differs dramatically across subcellular compartments. Disulfide-based triggers (green) require cytosolic GSH concentrations that are absent within endosomes. Thioketal-based triggers (orange) respond to ROS levels found primarily in the extracellular tumor microenvironment. Only diselenide-based systems (striped) respond to stimuli present in multiple compartments. The escape window (bracket) marks the narrow temporal period during which carriers must simultaneously destabilize the endosomal membrane and release their nucleic acid cargo.

The most widely exploited intracellular trigger is the reductive cleavage of disulfide bonds by glutathione (GSH). The standard rationale is straightforward: cytosolic GSH concentrations (2–10 mM) vastly exceed extracellular levels (2–20 µM), creating a sharp gradient that can be harnessed for selective intracellular release. However, this rationale conflates "intracellular" with "cytosolic." The endosomal and lysosomal lumens, where polyplexes spend the majority of their intracellular residence time, are oxidizing environments with GSH to oxidized glutathione (GSSG) ratios of approximately 1:1 to 3:1, dramatically lower than the cytosolic ratio of 30:1 to 100:1 (Fig. 4). Total glutathione may actually be higher in certain organellar compartments than the cytosol, but the biologically active reduced form (GSH) is not [101]. In the ER, for example, GSSG is retained and accumulated while GSH equilibrates freely across the membrane [102, 103]. Within endosomes and lysosomes specifically, the acidic, oxidizing environment disfavors reduction of GSSG to GSH, meaning that disulfide-containing polyplexes trapped in these compartments will not experience meaningful reductive cleavage.

This creates a tension for disulfide-based release systems. The standard design rationale assumes that productive GSH-triggered degradation requires the polyplex to first escape the endosome intact, as a complete, carrier-bound complex, into the reducing cytosol. But Sect. "The Mechanistic Basis of the Escape–Release Tradeoff" established that intact polyplex escape appears to be rare. The Rehman *et al.* live-cell imaging data showed that at the moment of productive release, polymer and nucleic acid are separately expelled through transient pores, and that only one or two endosomes per cell contribute to escape. If the polyplex dissociates during pore-mediated escape rather than escaping intact, the cytosolic GSH trigger may never encounter the disulfide bonds in the carrier backbone as designed.

The same spatial logic applies to esterase-responsive systems. Cytosolic esterases can hydrolyze ester-containing polymers to reduce charge and promote cargo release, but endosomal and lysosomal esterases (primarily acid hydrolases like cathepsins) operate through distinct mechanisms and substrate specificities. Endosomal and cytosolic esterases have different substrate specificities, so a polymer designed for cleavage by cytosolic carboxylesterases may remain intact in the endosome. Conversely, a polymer that is cleaved by endosomal acid hydrolases may release cargo before escape has occurred, exposing free RNA to endosomal nucleases.

By contrast, reactive oxygen species (ROS) and certain extracellular matrix enzymes (such as matrix metalloproteinases) operate in the extracellular or pericellular space, meaning that carriers responsive to these triggers can begin degradation *before* endocytosis. This shifts the release event upstream in the delivery pathway, a fundamentally different temporal relationship to escape than cytosol-dependent triggers.

These spatial distinctions motivate the organization of this section (Fig. 4). We evaluate non-pH stimulus-responsive strategies not by their chemistry but by where the trigger acts relative to the endosome: cytosol-dependent triggers that require intact polyplex escape (Sect. "Cytosol-Triggered Release: Do Bioreducible Carriers Resolve the Tradeoff?"), microenvironment-dependent triggers that act before or during endocytosis (Sect. "Microenvironment-Triggered Release: A Different Spatial Relationship to Escape"), and dual-trigger designs that attempt to align escape and release through orthogonal stimuli operating in the same compartment (Sect. "Dual-Trigger and Sequential-Trigger Designs: Toward Kinetic Alignment of Escape and Release"). For each category, we ask whether the spatial and temporal alignment of the trigger with the escape event genuinely decouples the tradeoff or merely shifts the failure mode from one step to another. Table 2 summarizes each trigger class by where it acts, its advantage for the tradeoff, and its characteristic failure mode.

**Table 2:** Non-pH Stimulus-Responsive Strategies Evaluated Through the Escape–Release Tradeoff

Trigger Chemistry	Stimulus	Where It Acts	Spatial Requirement for Productive Release	Advantage for Tradeoff	Characteristic Failure Mode	Representative Systems
Disulfide/GSH	Thiol-disulfide exchange by cytosolic GSH (2–10 mM)	Cytosol only. Endosomal GSH:GSSG ratio (~ 1:1–3:1) is insufficient for cleavage	Requires intact polyplex to escape endosome into reducing cytosol—a rare event (Sect. "The Mechanistic Basis of the Escape–Release Tradeoff")	Decouples escape (cationic charge retained through trafficking) from release (reductive degradation post-escape); reduces carrier toxicity via degradation into low-MW fragments	**Redundant trigger**: if polyplex dissociates during pore-mediated escape, cytosolic GSH encounters carrier fragments, not intact disulfides. Release mechanism may not be activated as designed	Disulfide-crosslinked PEI [104].; bioreducible PBAEs [105].; disulfide-linked PAMAM [90].; mixed reducible/non-reducible PLys [35].
Thioketal/ROS	Oxidative cleavage by H₂O₂, ·OH, O₂⁻, ^1^O₂ (tumor: 50–100 µM H₂O₂)	Tumor microenvironment (extracellular). Intracellular ROS levels generally insufficient except in highly stressed cells	Does NOT require intact polyplex escape—degradation can begin before endocytosis	Bypasses the spatial paradox of cytosol-dependent triggers; carrier degradation occurs upstream of endocytosis	**Premature release**: ROS-triggered degradation before endocytosis strips cationic charge needed for membrane destabilization, compromising escape. Free RNA exposed to endosomal nucleases	Poly(amino thioketal) [106].; CA-PLL-TK [107].; TKPFH [108].; TK-PEI + photosensitizer [109].
Boronic acid/ROS (charge-reversal)	ROS-triggered oxidation of boronic acid → elimination cascade → quaternary ammonium converts to tertiary amine → self-catalyzed ester hydrolysis → polyanion	Cytosol (intracellular H₂O₂) and potentially tumor microenvironment	Partially compartment-independent, but ROS exposure timing relative to escape is critical	Charge inversion actively expels cargo (cation → anion) rather than merely weakening binding; separates escape function (cationic) from release function (anionic) mechanistically	**Kinetic misalignment**: the same ROS that triggers charge reversal is present extracellularly, so degradation may begin before escape is complete. Rate of boronic acid oxidation *vs.* rate of endosomal escape determines outcome	B-PDEAEA [110].; lipid-coated B-PDEAEA [111].
Diselenide/dual (ROS + GSH)	Cleavable by BOTH oxidation (ROS) and reduction (GSH). Se–Se bond energy ~ 172 kJ/mol *vs.* S–S ~ 240 kJ/mol	Multiple compartments: cytosol (GSH), tumor microenvironment (ROS), and potentially endosomes (if membrane damage permits GSH leakage)	Less spatially constrained than disulfide or thioketal alone—can respond to stimuli in multiple compartments	Dual responsiveness provides multiple activation opportunities; lower bond energy than disulfide enables cleavage under milder conditions	**Ambiguous sequencing**: unclear whether ROS and GSH triggers operate sequentially (one for escape, one for release) or redundantly (both contributing to the same degradation event). No evidence of true sequential operation exists	OEI800-SeSe [112].; DSe-PEI-F [113].; diselenide PBAEs [114].
Esterase	Hydrolytic cleavage by esterases (cytosolic or endosomal, with different substrate specificities)	Endosomal (cathepsins, acid hydrolases) AND cytosolic (carboxylesterases) — but enzyme populations differ between compartments	Depends on design: endosomal esterase cleavage requires no escape; cytosolic esterase cleavage requires escape	Endosomal esterase activity could enable release concurrent with escape (within the escape window); PBAE hydrolysis (t₁/₂ ~ 4–6 h at pH 7.4) provides built-in temporal delay	**Premature endosomal release** (if endosomal esterases cleave too early) OR **delayed cytosolic release** (if cytosolic esterases are too slow). PBAE hydrolytic half-life may be too slow relative to the ~ 10–30 min escape window	PBAEs (general class) [115, 116].; ester-containing PAMAM variants [117, 118].

### Cytosol-Triggered Release: Do Bioreducible Carriers Resolve the Tradeoff?

The most widely investigated non-pH stimulus-responsive strategy for polymeric nucleic acid delivery is the incorporation of reduction-sensitive disulfide bonds into carrier backbones, crosslinkers, or shell-core linkages. The design logic is appealing: disulfide bonds remain stable in the mildly oxidizing extracellular environment (GSH 2–20 µM) but are rapidly cleaved by thiol-disulfide exchange upon exposure to cytosolic glutathione (2–10 mM), triggering carrier degradation and cargo release [119, 120]. In principle, this chemistry decouples the escape and release events. The carrier retains its cationic, membrane-destabilizing architecture throughout endosomal trafficking, then disassembles selectively in the reducing cytosol. Bioreducible polycations including disulfide-crosslinked PEI [121–123], disulfide-containing poly(amido amine) [124] and disulfide-linked poly(β-amino esters) [105] have demonstrated improved transfection efficiency and reduced cytotoxicity compared to their non-degradable counterparts across a range of nucleic acid cargoes including siRNA, mRNA, pDNA, and shRNA [125].

However, evaluating these systems through the escape–release tradeoff reveals a critical spatial assumption that is rarely examined. As Sect. "The Spatial Mismatch Problem" established, productive GSH-triggered disulfide cleavage requires the polyplex to first escape the endosome intact into the reducing cytosol. The endosomal and lysosomal lumens are oxidizing environments with GSH:GSSG ratios of only 1:1 to 3:1 [126], meaning that disulfide bonds in polyplexes trapped within endosomes, where polyplexes spend the vast majority of their intracellular residence time, will not undergo meaningful reductive cleavage. Yet Sect. "The Mechanistic Basis of the Escape–Release Tradeoff" and the preceding discussion in this section establish that intact polyplex escape is exceedingly rare, and that polymer and nucleic acid dissociate during the pore-mediated escape event itself rather than after cytosolic release [29]. If the polyplex has already dissociated at the moment of escape, the cytosolic GSH trigger encounters carrier fragments rather than intact disulfide-crosslinked complexes, and the designed release mechanism becomes functionally redundant.

This paradox raises a question: if bioreducible carriers demonstrably improve transfection relative to non-degradable analogs, yet the intended cytosolic release mechanism may rarely be activated as designed, what accounts for their efficacy? Several non-exclusive explanations are worth considering.

First, the reduced cytotoxicity of degradable fragments, rather than enhanced cargo release itself, may be the primary advantage. Cationic polymers containing disulfide bonds are degraded into lower-molecular-weight cationic fragments and sulfhydryl groups in reducing environments, decreasing membrane toxicity relative to non-degradable polycations of equivalent charge density [125].

Second, partial disulfide reduction may occur within late endosomes themselves, where some GSH is present even at unfavorable GSH:GSSG ratios, particularly if endosomal membrane damage permits limited cytosolic GSH leakage during the escape process. Wang *et al.* note that while most subcellular compartments maintain GSH in a highly reduced state, total glutathione distribution is heterogeneous, and the picture within maturing endosomes during active membrane destabilization remains poorly characterized [29].

Third, thiol-disulfide exchange at the endosomal membrane surface, between carrier disulfides and membrane-associated thiols, may contribute to both membrane destabilization and partial carrier disassembly. This has been suggested for disulfide-containing lipid systems where thiol-blocking agents significantly reduced cellular uptake [127]. Such a mechanism would be spatially coincident with escape: the escape event itself initiates partial carrier degradation through membrane thiol exchange rather than bulk cytosolic GSH reduction.

The functional consequence is that intermediate decomplexation kinetics, neither too fast nor too slow, consistently outperform extremes, as Hwang, Kang, and Bae showed for mixed reducible/non-reducible PLys polyplexes (Sect. "Why Escape and Release Are Inherently Opposed") [35]. In the context of bioreducible carriers, this observation takes on additional significance. It suggests that the rate of carrier degradation matters more than the location of the trigger, and that systems designed for instantaneous cytosolic disassembly may actually release cargo too rapidly for productive RISC loading or ribosomal engagement, particularly for larger mRNA cargo where some degree of continued association may facilitate cytosolic transport.

The net assessment is that disulfide-based bioreducible carriers represent a meaningful improvement over non-degradable polycations, but their mechanism of action likely differs from the clean cytosol-selective release narrative that dominates the literature. The reduction in carrier toxicity, rather than spatial selectivity of cargo release, may be the dominant contributor to their improved therapeutic index. These systems do not fully resolve the fundamental escape–release tradeoff identified in Sect. "The Mechanistic Basis of the Escape–Release Tradeoff". The disulfide trigger provides a temporal delay between escape and release, but this delay is productive only to the extent that intact polyplexes actually reach the cytosol, an event that, based on current evidence, occurs far less frequently than the field assumes.

### Microenvironment-Triggered Release: A Different Spatial Relationship to Escape

The spatial paradox confronting cytosol-dependent triggers, that productive release requires an intact polyplex escape event that rarely occurs, motivates a fundamentally different design philosophy: placing the degradation trigger *upstream* of endocytosis rather than downstream. Reactive oxygen species and certain extracellular matrix enzymes such as matrix metalloproteinases (MMPs) are elevated in the tumor microenvironment at concentrations sufficient to initiate carrier degradation before or during cellular uptake, shifting the release event to the extracellular or pericellular space. This temporal repositioning means that microenvironment-triggered carriers do not depend on intact polyplex escape into the cytosol, a critical distinction from the disulfide-based systems evaluated in Sect. "Cytosol-Triggered Release: Do Bioreducible Carriers Resolve the Tradeoff?".

#### ROS-cleavable Carriers Exploit Tumor Oxidative Stress

The tumor microenvironment is characterized by elevated H₂O₂ concentrations (50–100 µM compared to much lower levels in normal tissue), driven by mitochondrial dysfunction, NADPH oxidase activity, and metabolic reprogramming [125]. Thioketal linkages are the most extensively investigated ROS-responsive group for polymeric nucleic acid delivery, sensitive to a broad spectrum of ROS including H₂O₂, ·OH, O₂⁻, and ^1^O₂ while remaining stable in enzymatic, acidic, and alkaline environments [106, 125]. Shim and Xia first demonstrated the successful application of thioketal-containing poly(amino thioketal) for delivering nucleic acids to cancer cells, exploiting the ROS-triggered cleavage of thioketal into acetone and thiol fragments to achieve carrier degradation [106]. Subsequent developments have expanded the thioketal polyplex repertoire: Wan *et al.* prepared a polycationic poly(L-lysine) backbone linked with thioketal and cis-aconitate (CA-PLL-TK) for co-delivering siFGL1 and siPD-L1, where thioketal cleavage in the ROS-rich tumor environment triggered carrier degradation and siRNA release [107]. Yang *et al.* constructed thioketal-crosslinked fluorinated PEI (TKPFH) for co-delivering shGPX4 and shMTHFD2 plasmids, where ROS-triggered thioketal cleavage released the shRNA payloads and the resulting GPX4 knockdown further elevated intracellular ROS, creating a feed-forward loop that amplified both carrier degradation and ferroptotic cell death [108].

However, evaluating these systems through the escape–release tradeoff reveals a critical limitation: the spatial advantage of extracellular ROS triggering comes at the cost of *temporal*misalignment with the escape event. If ROS-mediated carrier degradation initiates before endocytosis, the partially degraded carrier may lose the cationic charge density required for membrane destabilization and endosomal escape. A carrier that releases cargo prematurely, before escape has occurred, exposes free nucleic acid to endosomal nucleases, producing the same failure mode as inadequate complexation. The thioketal systems cited above address this through design strategies that attempt to preserve escape function: CA-PLL-TK incorporates cis-aconitate for pH-triggered lysosomal escape independent of cationic charge [107], while TKPFH uses an outer hyaluronic acid shell for CD44-targeted uptake followed by intracellular ROS triggering [108]. Wang *et al.* took a different approach, co-delivering a photosensitizer (pheophytin a) with p53 pDNA in thioketal-crosslinked PEI, using far-red light irradiation to actively generate ROS after endocytosis, thereby controlling the *timing*of thioketal cleavage to occur within the endosome rather than in the extracellular space [109]. This photo-triggered strategy converts an extracellular trigger into an intracellular one, but requires external irradiation and sacrifices the passive microenvironment-responsiveness that motivated the approach. This design represents a form of photochemical internalization (PCI), a broader class of externally-triggered escape strategies in which co-delivered photosensitizers generate reactive species upon light exposure to disrupt endosomal membranes on demand [128]. A related physical-trigger approach uses ultrasound and microbubbles (sonoporation) to induce transient membrane pores through acoustic cavitation [129]. The role of physical triggers of this kind is best understood as complementary to intrinsic-trigger chemistry: physical stimuli offer on-demand spatiotemporal control that intrinsic chemistries cannot match, while intrinsic chemistries provide the compartment-selective release logic that physical stimuli largely lack; hybrid designs like Wang *et al.* exploit both.

A further consideration specific to ROS-responsive systems is the heterogeneity of tumor ROS concentrations. Not all cancer cells produce H₂O₂ at levels sufficient to cleave thioketal linkages, and the uneven spatial distribution of ROS within a single tumor means that carrier degradation, and therefore cargo release, will vary substantially across the tumor cell population [125]. This stands in contrast to cytosolic GSH, which, despite the compartmentalization issues discussed in Sect. "The Spatial Mismatch Problem", is relatively uniform within the cytoplasm of individual cells once escape has occurred. The field has partially addressed this through active ROS generation strategies. Wang *et al.* and Deng *et al.* have co-loaded photosensitizers (chlorin e6, pheophytin a) with nucleic acid cargo in thioketal-containing carriers, using light-triggered ^1^O₂ production to ensure sufficient local ROS concentrations for complete carrier degradation [109, 130, 131]. While effective in preclinical models, this approach introduces a dependency on external light penetration that limits applicability to superficial or surgically accessible tumors.

The net assessment for microenvironment-triggered systems is therefore mixed. The spatial advantage, bypassing the requirement for intact polyplex escape, is genuine and addresses the central weakness of cytosol-dependent triggers. However, this advantage introduces a new tradeoff: premature release before escape, rather than insufficient release after escape. The design challenge shifts: rather than releasing cargo after escape, the problem becomes preserving enough carrier integrity for escape to occur before the microenvironmental trigger degrades it. This is, in essence, a kinetic alignment problem, and it motivates the dual-trigger approaches discussed in Sect. "Dual-Trigger and Sequential-Trigger Designs: Toward Kinetic Alignment of Escape and Release".

### Dual-Trigger and Sequential-Trigger Designs: Toward Kinetic Alignment of Escape and Release

The preceding sections establish that single-trigger strategies each encounter a characteristic failure mode: cytosol-dependent triggers (Sect. "Cytosol-Triggered Release: Do Bioreducible Carriers Resolve the Tradeoff?") require an intact polyplex escape event that rarely occurs, while microenvironment-dependent triggers (Sect. "Microenvironment-Triggered Release: A Different Spatial Relationship to Escape") risk premature cargo release before escape is complete. A logical design response is to employ two orthogonal stimuli, one to drive or preserve endosomal escape and a second to trigger cargo release, operating in sequence within the same delivery pathway. In principle, such dual-trigger systems could achieve what neither single trigger accomplishes alone: maintaining carrier integrity through uptake and escape, then initiating rapid degradation once the polyplex reaches an environment where release would be productive.

#### Diselenide Linkages Offer Intrinsic Dual-responsiveness

Unlike disulfide bonds, which respond primarily to GSH, diselenide bonds (Se–Se) can be cleaved by both reductive (GSH) and oxidative (ROS, H₂O₂) stimuli, owing to the lower bond energy of Se–Se (~ 172 kJ/mol) compared to S–S (~ 240 kJ/mol) and the larger atomic radius and weaker electronegativity of selenium [125, 132]. This dual sensitivity has been exploited in several polymeric nucleic acid delivery systems. Cheng *et al.* synthesized diselenide-crosslinked oligoethylenimine (OEI800-SeSe) that exhibited reductive sensitivity comparable to its disulfide analog while maintaining low cytotoxicity and improved transfection [112]. Deng *et al.* first applied the ROS sensitivity of diselenide in nucleic acid delivery by crosslinking low-molecular-weight PEI with a diselenide linker and fluorocarbon modification; in the high-ROS environment of cancer cells, diselenide oxidation triggered polymer degradation and rapid pDNA release [113]. He *et al.* constructed layered "nano-onion" structures (UCNOs) with a diselenide-crosslinked PEI middle layer loaded with siRNA, where NIR-triggered ROS generation from a photosensitizer core cleaved the diselenide bonds and released the therapeutic payload [133]. More recently, Jiang *et al.* developed a library of diselenide-containing PBAEs for mRNA delivery that exhibited dominant spleen-targeted delivery in mice, contrasting with the lung tropism typical of conventional PBAEs, suggesting that the diselenide moiety alters not only release kinetics but also biodistribution [114].

However, the critical question for dual-responsive diselenide systems is whether the two triggers operate sequentially, with one driving escape and the other driving release, or merely redundantly, with both contributing to the same degradation event at the same time. The distinction matters for the escape–release tradeoff. If diselenide cleavage by endosomal or microenvironmental ROS initiates carrier degradation before escape, the system encounters the same premature release problem identified for thioketal carriers in Sect. "Microenvironment-Triggered Release: A Different Spatial Relationship to Escape". If cytosolic GSH cleaves the diselenide after escape, the system encounters the same spatial paradox as disulfide carriers in Sect. "Cytosol-Triggered Release: Do Bioreducible Carriers Resolve the Tradeoff?", requiring intact polyplex escape that Sect. "The Mechanistic Basis of the Escape–Release Tradeoff" established is rare. True sequential operation would require that the ROS trigger partially destabilizes the carrier to facilitate escape (without fully releasing cargo), and the GSH trigger subsequently completes degradation to liberate intact nucleic acid. Whether any existing diselenide polyplex system achieves this sequential behavior, rather than simultaneous or condition-dependent responsiveness, has not been demonstrated. The field currently lacks assays capable of resolving the temporal sequence of carrier degradation relative to endosomal escape at the single-endosome level, a limitation we return to in Sect. "Measuring Escape and Release: The Assay Gap".

#### Charge-reversal Systems Offer an Alternative Dual-trigger Logic

ROS-triggered positive-to-negative charge conversion, exemplified by boronic acid-containing poly[(2-acryloyl)ethyl(p-boronic acid benzyl)diethylammonium bromide] (B-PDEAEA), represents a mechanistically distinct approach [110]. In this system, the cationic quaternary ammonium groups condense DNA and facilitate cellular uptake. Upon exposure to intracellular H₂O₂, oxidation of the boronic acid moiety triggers elimination of p-quinone methide, converting quaternary ammonium to tertiary amine, which undergoes self-catalyzed ester hydrolysis to yield negatively charged poly(acrylic acid). The complete charge inversion, from polycation to polyanion, actively expels the nucleic acid cargo rather than merely weakening electrostatic binding. This is conceptually compelling because it separates the escape function (cationic carrier destabilizes endosomal membrane) from the release function (charge reversal ejects cargo) through different chemical events triggered by the same stimulus (ROS). Xiang *et al.* further enhanced this system by coating B-PDEAEA polyplexes with cationic lipids that improved serum stability, lysosomal escape, and amplified intracellular ROS generation to accelerate the charge-reversal process [111].

Yet even this design does not fully resolve the kinetic alignment problem. The ROS trigger that initiates charge reversal is the same oxidative environment present in the tumor microenvironment, meaning that degradation could begin extracellularly or within early endosomes, before the cationic carrier has completed its membrane-destabilizing function. The rate of boronic acid oxidation relative to the rate of endosomal acidification and escape determines whether cargo release is productive or premature, and this kinetic relationship will vary with tumor type, ROS concentration, and endosomal trafficking dynamics.

**Fig. 5:**
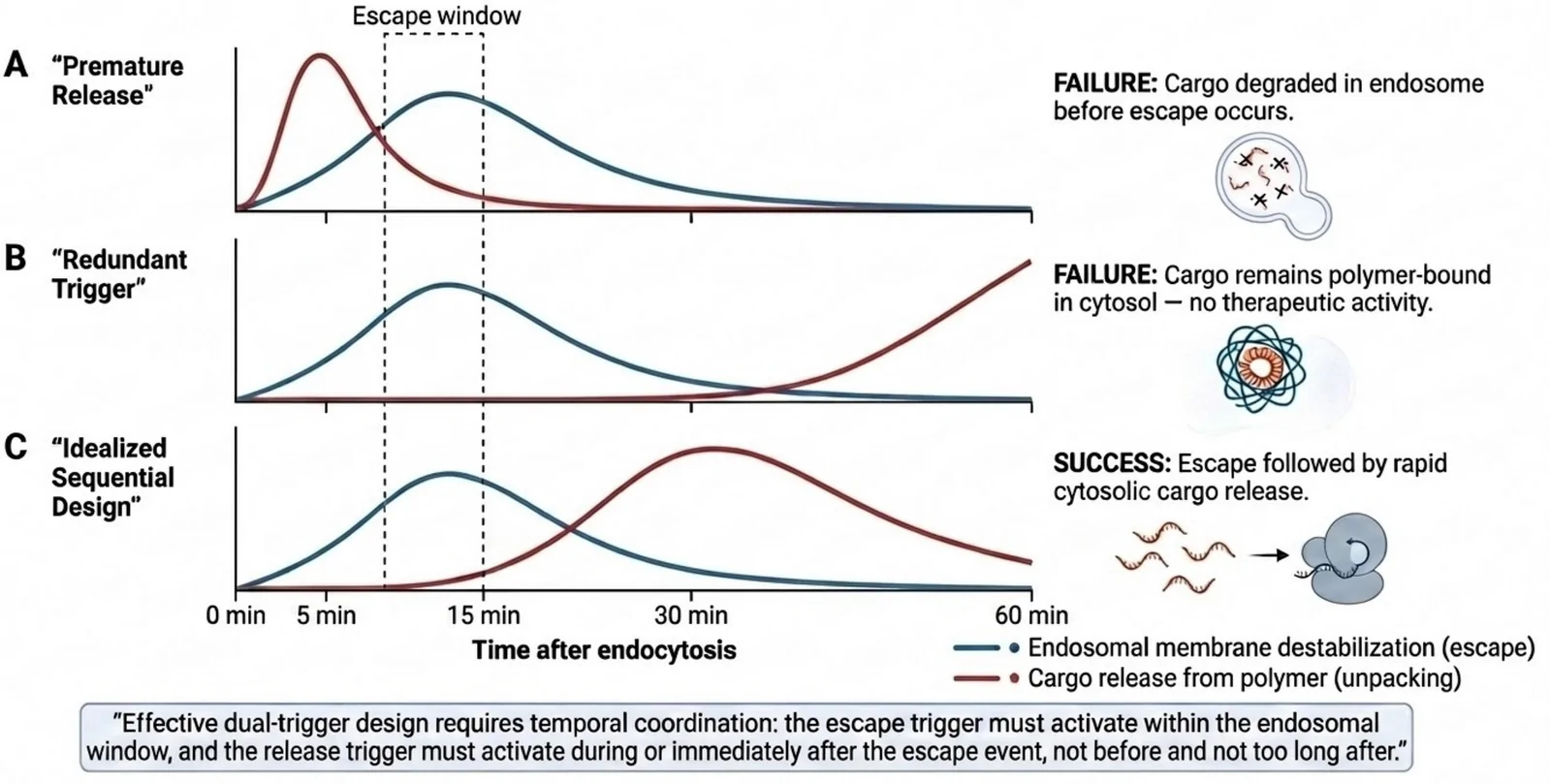
The kinetic alignment problem for dual-trigger carrier designs. The escape window (bracketed, 5–15 min after endocytosis) reflects the interval during which productive endosomal escape has been observed by live-cell imaging of nucleic acid delivery, with escape events concentrated in the transition between early and maturing endosomes. (**A**) Premature release: microenvironment-triggered release occurs before the carrier escapes the endosome, leading to cargo degradation. (**B**) Redundant trigger: cytosol-triggered release occurs too slowly after escape, leaving cargo therapeutically unavailable. (**C**) Idealized sequential design: escape and release are temporally coordinated, with release initiating immediately upon cytosolic entry.

The emerging picture from dual-trigger designs reinforces the point that the escape–release tradeoff cannot be resolved by chemistry alone. Even systems incorporating two orthogonal triggers must contend with the temporal alignment of those triggers relative to the endosomal maturation window during which productive escape has been observed to occur (Fig. 5). Live-cell imaging of siRNA release from lipid-formulated carriers has estimated this window at approximately 5–15 min after endocytosis, with escape events concentrated in the transition between early and maturing endosomes [11, 14, 134]. The window is not sharply bounded and shifts with cell type and carrier chemistry, but the constraint on trigger timing is substantial: a release chemistry with kinetics slower than the escape window itself will act on cargo that has already been committed to lysosomal degradation rather than delivered. The most promising approaches may be those that exploit the escape event itself as the trigger for release, similar to the pH-triggered conformational change in influenza hemagglutinin (HA2) that simultaneously drives membrane fusion and viral uncoating [135]. Systems in which the physical act of membrane disruption, pore formation, or pH neutralization upon cytosolic entry initiates a conformational or chemical change that liberates cargo would achieve the same coupling synthetically. The charge-altering releasable transporters (CARTs) discussed in Sect. "pH-responsive and charge-shifting polymers as escape–release coupling devices" approach this ideal through self-immolative degradation triggered by the shift from endosomal to cytosolic pH (t₁/₂ ≈ 2 min at physiological pH),⁹⁸ though even CARTs face the constraint that their degradation kinetics must be fast enough to release cargo before cytosolic nucleases act but slow enough to maintain carrier integrity during trafficking. Whether polymer engineering can achieve the kinetic precision required to match release timing to the stochastic, low-frequency escape events described in Sect. "The Mechanistic Basis of the Escape–Release Tradeoff" remains an open question, and one that demands the improved assay capabilities discussed in Sect. "Measuring Escape and Release: The Assay Gap".

## Polymer Architecture and Formulation: Structural Approaches to Decoupling Escape from Release

The strategies reviewed in Sects. "pH-responsive and charge-shifting polymers as escape–release coupling devices" and "Non-pH Stimulus-Responsive Strategies: Do Alternative Triggers Resolve the Tradeoff?" attempt to decouple escape from release through chemistry: pH-responsive charge shifts, orthogonal triggers, or dual-stimulus designs. A complementary approach is to decouple them through *structure*, by physically separating the polymer domains responsible for membrane destabilization from those responsible for nucleic acid condensation. Architecture refers to the polymer's covalent organization (backbone topology, block sequence, molecular weight, branching), while formulation refers to its supramolecular presentation (micelle assembly, N/P ratio, PEG density). Both levels influence the escape-release balance, but they do so through different levers [4, 136].

### Why Architecture Matters for the Tradeoff

In single-component polycations such as linear PEI or branched PEI, all ionizable amines serve dual roles: they condense nucleic acid and they destabilize endosomal membranes. This dual function is the structural origin of the coupling described in Sect. "The Mechanistic Basis of the Escape–Release Tradeoff". A polymer cannot selectively direct some of its amines toward the membrane and others toward the cargo, because all amines are distributed along the same backbone without spatial isolation [137]. Funhoff *et al.* provided direct evidence of this limitation: pDAMA, a polymer with strong pH 5–7 buffering from two tertiary amine groups per repeat unit, produced poor transfection despite its buffering capacity. Adding a membrane-disruptive peptide significantly improved transfection without affecting cytotoxicity, confirming that buffering and membrane disruption are independent requirements that single-component architectures cannot simultaneously optimize [137].

Branching introduces partial improvements. In PBAEs, increasing the degree of branching (from linear to bifunctional to trifunctional to tetrafunctional) improved endosomal disruption as measured by a Gal8-based assay, with the improvement attributed to altered polyplex geometry and increased surface amine density [138]. However, branching does not spatially isolate escape and condensation functions. It increases the efficiency of both simultaneously, without providing independent control over either.

Lipid nanoparticles offer an instructive contrast. In current structural models, LNP-encapsulated RNA is complexed with ionizable lipid within internal inverted-micelle-like domains, while a distinct population of ionizable lipid at the particle surface drives interaction with the endosomal membrane [18]. The membrane-active surface population and the cargo-complexing interior population are the same chemical species, but they occupy spatially distinct positions within the same particle, and the surface population has no direct access to the interior-complexed cargo. This division of labor does not eliminate the thermodynamic tradeoff, but it does mitigate it, and it may explain why the tradeoff is most acute in structurally simple, homogeneous polycations where every ionizable amine serves both roles at once. The block and layered architectures reviewed below can be understood as attempts to recover, within polymeric carriers, the spatial segregation that LNP architecture achieves through composition alone.

### Block Copolymers: Spatial Segregation of Escape and Release Domains

Block copolymer architectures address the tradeoff more directly by assigning escape and condensation to separate covalent domains within the same polymer chain.

#### Diblock Copolymers Provide the Proof of Concept

The seminal system in this class is the poly(2-(dimethylamino)ethyl methacrylate) (pDMAEMA)-b-(DMAEMA-co-PAA-co-BMA) diblock (D-b-DBP), which places nucleic acid condensation in the pDMAEMA block and pH-responsive membrane destabilization in the core-forming block containing DMAEMA, propylacrylic acid (PAA), and butyl methacrylate (BMA) [139]. Under optimal conditions, these micelles delivered siRNA with over 90% target gene knockdown, attributed to simultaneous membrane-lytic activation and siRNA release as the block structure disassembled at endosomal pH. Manganiello *et al.* subsequently tuned this architecture by systematically varying the BMA content (20–70 mol%) in a simplified pDMAEMA-b-(DEAEMA-co-BMA) system synthesized by reversible addition-fragmentation chain-transfer (RAFT) polymerization [140]. The BMA content (20–70 mol%) controls the pH threshold at which the hydrophobic core disassembles and exposes membrane-active segments. The 40% BMA composition, which showed the sharpest pH transition within the early-to-mid endosomal window, produced up to 20-fold more transfection than branched PEI in monocyte cell lines [141]. Adding propylacrylic acid to the escape block creates an even sharper membrane-disruptive transition, achieving over 90% siRNA-mediated knockdown through simultaneous membrane lysis and siRNA release as the block structure disassembles at endosomal pH [142]. This simultaneous activation is advantageous for siRNA (which requires rapid cytosolic release) but potentially limiting for mRNA, where some continued polymer association may protect the transcript from cytoplasmic RNases immediately after escape. The diblock architecture thus demonstrates that spatial separation of escape and condensation domains is achievable, but the two events remain temporally coupled because both are triggered by the same pH-driven disassembly.

#### Triblock Copolymers Achieve Full Spatial and Functional Segregation

The triblock ABC architecture physically isolates the membrane-interactive block from the nucleic acid-binding block by placing them in separate structural compartments. Miyata, Fukushima, and Kataoka demonstrated this with the PEG-PAsp(DET)-PLys system: DNA binds exclusively to the PLys core, while PAsp(DET) remains free in the intermediate shell [143]. The PAsp(DET) unit undergoes selective double protonation at endosomal pH (~ 5.5) rather than single protonation at physiological pH, generating enhanced membrane-disruptive charge only when escape is needed. This architecture produced an order of magnitude more transfection than a PEG-PLys diblock control with identical PLys block length, and was validated *in vivo* by EGFP expression in pancreatic tumor tissue after intravenous injection [143].

The functional advantage of this spatial segregation was confirmed by Kim *et al.* using FRET to track polyplex behavior after escape. In the triblock system, the PEG corona prevented irreversible cytosolic aggregate formation, a post-escape function unavailable to non-PEGylated diblock controls, which immediately formed large aggregates in serum that trapped cargo in functionally inert complexes [144]. The Lac-PEG-PSAO-PAMA three-layered system confirmed the same principle through pharmacological dissection: nigericin (which inhibits endosomal acidification) significantly decreased transfection, whereas hydroxychloroquine (an independent endosomolytic agent) produced no further improvement, indicating that the free PSAO intermediate block had already saturated the available escape pathway [145].

The triblock architecture represents the most direct structural solution to the escape-release tradeoff because it separates the two functions into independently optimizable domains. The condensation block can be tuned for cargo binding strength without affecting escape, and the escape block can be tuned for membrane destabilization without affecting cargo retention.

#### Bottlebrush Conjugates Offer a Non-cationic Alternative

An entirely different approach to the tradeoff is to bypass electrostatic condensation altogether. Zhang, Dong, and colleagues created bottlebrush polymer-antisense oligonucleotide conjugates (pacDNA) in which the nucleic acid is covalently grafted to the polymer side chains rather than electrostatically condensed. The escape mechanism is not fully characterized but has been attributed to the steric bulk and high local concentration of the bottlebrush within the endosome, which may physically disrupt membrane integrity through crowding rather than electrostatic destabilization [146]. These conjugates achieved airway epithelial uptake and gene silencing *in vivo* through steric selectivity of the cylindrical bottlebrush architecture rather than cationic charge, including endosomal escape without any free amine groups to compete between buffering and cargo binding [147]. This non-cationic architecture completely avoids the protonation-driven coupling that creates the tradeoff, though it requires covalent conjugation chemistry and is restricted to nucleic acids compatible with such attachment.

### Formulation-Level Control

Architecture defines the intrinsic capabilities of a polymer, but formulation determines whether those capabilities are expressed in a biological environment. Two formulation variables have the most direct impact on the escape-release balance.

#### PEGylation Creates a Second Tradeoff Layered on the First

PEG shields are essential for systemic circulation but physically block the encounter between pH-responsive escape domains and the endosomal membrane. This is a formulation-level conflict: the same PEG that prolongs circulation inhibits escape. Miteva *et al.* showed that in the pDPB mixed micelle system, intermediate PEG density (50 mol% PEGylated) outperformed both extremes: 100% non-PEGylated micelles had poor serum stability, while 100% PEGylated micelles had poor escape [148]. FRET quantification of intracellular siRNA unpackaging confirmed that 50D/20 k PEG micelles achieved higher cytoplasmic bioavailability than fully PEGylated micelles despite lower cellular uptake, demonstrating that the uptake-release relationship is not monotonic with PEG content [148]. Acid-labile PEG shedding resolves this by temporally separating the functions: stable PEG protects the carrier in circulation, and pH-triggered PEG removal restores membrane activity in the late endosome [149, 150]. This strategy directly parallels the sequential trigger logic discussed in Sect. "Dual-Trigger and Sequential-Trigger Designs: Toward Kinetic Alignment of Escape and Release".

#### PEG Alternatives Address Stealth Without Inheriting PEG's Limitations

The reliance on PEG as the default stealth polymer is worth interrogating. PEG faces documented translational constraints, including accelerated blood clearance on repeat dosing driven by anti-PEG antibodies and structural rigidity in some architectures. Several backbone chemistries serve the same stealth function without these concerns. Polysarcosine, a poly(N-substituted glycine), provides similar hydration and steric shielding with reduced immunogenicity [151]. Polyoxazolines (POx) offer tunable amphiphilicity and comparable pharmacokinetics with lower observed anti-carrier immune activation [152, 153]. Zwitterionic polymers (poly(carboxybetaine), poly(sulfobetaine), poly(phosphorylcholine)) exploit charge-balanced hydration for stealth performance that in some studies exceeds PEG [154]. Most polymeric nucleic acid delivery systems developed with these alternatives pair them with independent escape-active blocks, echoing the diblock and triblock strategies of Sect. "Block Copolymers: Spatial Segregation of Escape and Release Domains". A more provocative direction would be a single polymer that combines corona stealth and endosomolytic function within the same architecture: zwitterionic polymers bearing pH-responsive protonatable groups, or polysarcosine-alkyl amphiphiles with intrinsic membrane activity, represent plausible routes to collapsing corona-*versus*-escape into one material. Whether such designs can match the tunability of separately-engineered stealth and escape blocks remains an open question, but the strategic value would be substantial: reducing the number of independently-optimized components in a formulation directly shortens the path from carrier design to clinical manufacturability.

#### N/P Ratio Controls Whether Architecture-encoded Triggers can Execute

Even a well-designed stimulus-responsive architecture will fail if the N/P ratio produces electrostatic binding energy that exceeds the driving force for triggered disassembly. Zhu *et al.* demonstrated this directly in a dual-responsive mPEG-b-PLA-PHis-ssPEI system: siRNA release reached 58.67% at N/P 6, and Bcl-2 silencing reached 85.45% in MCF-7 cells at N/P 6, but decreased significantly at N/P 10 and 15 despite higher cellular uptake [155]. The disulfide redox trigger and the PHis pH-responsive escape trigger were both fully activated at the higher N/P ratios, but the excess electrostatic binding prevented them from executing. N/P ratio is therefore not simply a condensation parameter but a formulation-level gatekeeper that determines whether architectural design features can function as intended. Formation pH is an additional formulation variable: polyplexes assembled at acidic pH where the polymer is fully protonated may adopt different condensation architectures than those formed at neutral pH, potentially affecting both stability during trafficking and release kinetics after escape [156, 157].

Sects. "pH-responsive and charge-shifting polymers as escape–release coupling devices" through "Polymer Architecture and Formulation: Structural Approaches to Decoupling Escape from Release" have examined pH-responsive chemistries, non-pH stimulus triggers, physical stimuli, and architectural strategies through the lens of the escape–release tradeoff. Table 3 summarizes each strategy class, its principal advantage for decoupling escape from release, its characteristic failure mode, its most suitable cargo, and the direction current work is pursuing to address its limitations. It is intended to be a comparative overview intended to highlight where each strategy sits within the escape–release framework, and to make visible that no single class currently resolves both sides of the tradeoff on its own.

**Table 3:** Polymeric nucleic acid delivery strategies evaluated through the escape–release tradeoff

Strategy	Trigger Mechanism/Site	Advantage for the Escape–Release Tradeoff	Characteristic Limitation	Suitable Cargo	Direction for Improvement
**pH-responsive polymers (**Sect. "pH-responsive polymers: tuning the activation zone of membrane activity"**)**	Amine protonation at endosomal pH drives membrane destabilization. PBAE/PDEAEMA-class polymers also deprotonate at cytosolic pH after escape	Well-characterized chemistry. A pKₐ in the 6–7 range provides selective endosomal activation and, for PBAEs, escape-coupled release through cytosolic-pH deprotonation	The cationic charge that drives escape also retains cargo binding. Polymers with a pKₐ above 7.4, such as PEI and PLys, derive little release benefit from cytosolic exposure	Any cargo; PBAE-class polymers are especially suited to siRNA and mRNA	Engineer pKₐ values to widen the escape–release window and pair pH-responsive polymers with charge-shifting chemistries
**Charge-shifting polymers (**Sect. "Charge-shifting polymers: programming release after entry"**)**	Cationic-to-neutral or cationic-to-anionic conversion occurs after endosomal entry, as in CARTs and boronic acid systems	Actively expels cargo through charge inversion rather than merely weakening polymer–cargo binding. Conceptually separates the escape and release functions	Trigger timing must align with endosomal escape. Premature charge conversion can eliminate membrane-disruptive activity	siRNA and mRNA	Sequentially couple escape and release triggers within a single polymer
**Disulfide/GSH-bioreducible polymers (**Sect. "Cytosol-Triggered Release: Do Bioreducible Carriers Resolve the Tradeoff?"**)**	Cytosolic glutathione-mediated thiol–disulfide exchange cleaves the polymer backbone	In principle, places the release trigger after endosomal escape and reduces carrier toxicity through backbone degradation	Requires intact polyplex escape, which is a rare event. The polymer and nucleic acid may dissociate during pore-mediated escape before cytosolic glutathione can act	pDNA, siRNA, and mRNA	Pair with pH-responsive escape triggers and investigate possible late-endosomal partial-reduction pathways
**Thioketal/ROS-responsive systems (**Sect. "Microenvironment-Triggered Release: A Different Spatial Relationship to Escape"**)**	Extracellular tumor-microenvironment ROS cleaves thioketal groups before endocytosis	Bypasses the spatial paradox associated with cytosolic triggers because carrier degradation begins before the polyplex enters the endosome	Premature release before endosomal escape can remove cationic charge and expose cargo to endosomal nucleases. ROS heterogeneity across patients and tumors also introduces variability	siRNA and mRNA for tumor delivery	Generate ROS actively through co-delivered photosensitizers and combine ROS responsiveness with intracellular escape triggers
**Boronic acid/charge-reversal systems (**Sect. "Dual-Trigger and Sequential-Trigger Designs: Toward Kinetic Alignment of Escape and Release"**)**	ROS-triggered oxidation converts quaternary ammonium groups to tertiary amines and subsequently to polyanions through self-catalyzed hydrolysis	The same stimulus preserves the cationic escape function initially and later triggers release through charge inversion using mechanistically distinct chemical events	Productive delivery depends on alignment between the ROS oxidation rate and the endosomal escape rate. Premature oxidation may cause early release, while tumor ROS variability introduces heterogeneity	pDNA and mRNA	Achieve tighter kinetic control and develop strategies that compensate for tumor ROS heterogeneity
**Diselenide/dual-trigger systems (**Sect. "Dual-Trigger and Sequential-Trigger Designs: Toward Kinetic Alignment of Escape and Release"**)**	Se–Se bonds are cleaved by both cytosolic glutathione through reduction and microenvironmental ROS through oxidation	Provides multiple activation opportunities across intracellular and extracellular compartments. The relatively low Se–Se bond energy enables cleavage under mild conditions	Trigger sequencing remains ambiguous. It is unclear whether ROS and glutathione act sequentially to support escape and release or redundantly to produce simultaneous degradation. Direct evidence of sequential operation is lacking	siRNA, mRNA, and pDNA	Develop assays that resolve trigger sequence and carrier behavior at the single-endosome level, as discussed in Sect. "Measuring Escape and Release: The Assay Gap"
**Esterase-responsive systems (**Sect. "Dual-Trigger and Sequential-Trigger Designs: Toward Kinetic Alignment of Escape and Release"**)**	Endosomal acid hydrolases or cytosolic carboxylesterases enzymatically hydrolyze ester bonds	PBAE hydrolysis provides a built-in temporal delay. Endosomal esterase activity may also permit cargo release concurrent with escape	Endosomal esterase cleavage may cause premature release, whereas cytosolic esterase kinetics may be too slow relative to the approximately 5–15-min escape window	pDNA and mRNA	Develop compartment-selective esterase substrates and tune hydrolysis half-lives
**Physical triggers: PCI and sonoporation (**Sect. "Dual-Trigger and Sequential-Trigger Designs: Toward Kinetic Alignment of Escape and Release"**)**	Photochemical internalization uses a light-activated photosensitizer to generate ROS and disrupt the endosomal membrane. Sonoporation uses ultrasound cavitation to create transient membrane pores	Provides on-demand spatial and temporal control, thereby decoupling escape timing from intracellular chemical conditions	Primarily addresses escape rather than release, meaning cargo may remain polymer-bound after membrane disruption. Limited tissue penetration also restricts use to accessible tumors and superficial tissues	siRNA and mRNA for accessible tumors, superficial tissues, and skin	Combine physical escape triggers with intrinsic release chemistries, such as thioketal groups paired with a photosensitizer
**Diblock copolymers (**Sect. "Block Copolymers: Spatial Segregation of Escape and Release Domains"**)**	Spatially separated cargo-binding and membrane-active blocks undergo pH-triggered disassembly that exposes the escape-active domain	Separates escape and cargo-binding domains within a single polymer chain and enables membrane disruption and cargo release at endosomal pH	Escape and release remain temporally coupled because both are initiated by the same pH event. This may be limiting for cargo that benefits from continued polymer association	siRNA, for which rapid release may be beneficial; potentially less optimal for mRNA, which may benefit from slower release and continued protection	Tune disassembly kinetics according to cargo-specific protection and release requirements
**Triblock copolymers (**Sect. "Block Copolymers: Spatial Segregation of Escape and Release Domains"**)**	An ABC architecture contains a PEG corona, an escape-active middle block, and a cargo-binding core. The middle block activates selectively at approximately pH 5.5	Provides spatial and functional segregation among independently tunable domains. The PEG corona may also prevent post-escape aggregation in the cytosol	Manufacturing is complex. The PEG dilemma remains because PEG improves circulation and stability but may shield the membrane-active escape domain	pDNA, siRNA, and mRNA	Use PEG alternatives, including polysarcosine, poly(2-oxazoline), and zwitterionic polymers, or introduce cleavable PEG strategies
**Bottlebrush conjugates (**Sect. "Block Copolymers: Spatial Segregation of Escape and Release Domains"**)**	Nucleic acids are covalently grafted to polymer side chains rather than electrostatically condensed. Steric crowding contributes to membrane disruption	Avoids protonation-driven coupling because free amines do not compete between buffering and cargo binding	Requires covalent conjugation chemistry and is restricted to nucleic acids compatible with attachment. The endosomal escape mechanism remains incompletely characterized	Antisense oligonucleotides and cholesterol-conjugated siRNA	Expand cargo compatibility and characterize the steric-crowding-mediated escape mechanism

## Measuring Escape and Release: The Assay Gap

If the coupling developed in Sects. "The Mechanistic Basis of the Escape–Release Tradeoff" through "Polymer Architecture and Formulation: Structural Approaches to Decoupling Escape from Release" is correct, then optimizing polymeric carriers requires assays capable of measuring both processes, ideally simultaneously and within the same endosomal compartment. The current toolkit falls short of this standard. Existing methods quantify either escape or release in isolation, and the field lacks a unified assay that can distinguish escaped-but-still-complexed cargo from escaped-and-free cargo in the cytosol.

### Galectin Recruitment: Quantifying Endosomal Disruption Without Cargo Information

The galectin family of β-galactoside-binding lectins has emerged as the most direct and quantitative reporter of endosomal membrane integrity. Galectin-8 (Gal8), normally diffuse in the cytosol, rapidly redistributes to the glycosylated inner leaflet of disrupted endosomal membranes, producing fluorescent puncta when expressed as a Gal8-YFP or Gal8-GFP fusion protein [25, 158]. Kilchrist *et al.* showed that Gal8 recruitment correlated strongly with siRNA bioactivity across a library of PEG-b-(DMAEMA-co-BMA) polymers of varied composition and molecular weight (r = 0.95, p < 10⁻^4^), substantially outperforming Lysotracker colocalization (r =  − 0.36, not significant), pH-dependent hemolysis (not significant), and cellular uptake (r = 0.73, p < 10⁻^3^) as predictors of functional delivery [25]. The assay achieved a Z′ factor of 0.6, qualifying it as excellent for high-throughput screening, and was further validated *in vivo* in orthotopic breast tumors. Herrera, Kim, and colleagues subsequently demonstrated that the Gal8-GFP reporter system could detect endosomal disruption events for LNP-encapsulated mRNA, with phytosterol-substituted LNPs producing up to tenfold more Gal8 puncta than standard cholesterol LNPs in live-cell imaging over 16 h [159]. Importantly, their colocalization analysis showed that Gal8 recruitment occurred preferentially at Rab7-positive late endosomes rather than EEA1-positive early endosomes, consistent with the escape window discussed in Sect. "The Mechanistic Basis of the Escape–Release Tradeoff".

Despite these strengths, galectin-based assays report only on membrane disruption events. They do not distinguish whether cargo within a disrupted endosome remains polymer-bound or has been released, nor do they measure how much nucleic acid reaches the cytosol in a functionally available state.

### Split Luciferase Complementation: Quantifying Cytosolic Access of Carrier Components

The SLEEQ assay addresses a complementary aspect of the problem. A HiBiT peptide is conjugated to the nanoparticle core via a disulfide linkage, while a LgBiT protein is expressed as a cytosolic actin fusion. Luminescence occurs only when HiBiT escapes the endosome and encounters cytosolic LgBiT, with femtomolar detection sensitivity [30]. Applied to pH-responsive nanoparticles with tunable disassembly pH, SLEEQ revealed that particles engineered to disassemble at pH 6.4 (early-to-late endosomal transition) achieved 10–15% escape efficiency, compared to less than 2% for particles disassembling at pH 5.6. This fivefold difference could not be resolved by the conventional calcein assay, which provides only a binary and subjective readout [30]. However, because SLEEQ tracks the polymer-conjugated peptide rather than the nucleic acid cargo, it measures carrier escape, not cargo release. A polyplex that escapes the endosome with its nucleic acid payload still fully condensed would produce identical SLEEQ signal to one that releases its cargo upon escape.

### FRET-based Unpacking Assays: Tracking Dissociation Without Spatial Resolution

Förster resonance energy transfer (FRET) between labeled polymer and labeled nucleic acid can report on polyplex dissociation in real time. When the FRET pair is in close proximity (i.e., the polyplex is intact), energy transfer occurs; upon unpacking, the FRET signal is lost. This approach has been applied using quantum dot–Cy5 labeling to compare unpacking kinetics across different polymer chemistries, and has revealed that polyplex disassembly and transfection efficiency are correlated [160, 161]. However, FRET measurements are typically performed as bulk population averages by confocal microscopy or flow cytometry and lack single-endosome resolution. They cannot determine whether unpacking occurs before, during, or after endosomal membrane disruption.

### Live-cell Imaging of the Escape Event Itself

Rehman and colleagues provided the most direct visualization of polyplex-mediated endosomal escape to date, using spinning-disk confocal microscopy to capture the process in real time in HeLa cells [29]. Their observations challenged the prevailing assumption that the proton sponge effect causes complete endosomal lysis. Instead, they observed local membrane rupture through which nucleic acids were discharged in a rapid, directional manner, within seconds, while the endosomal compartment itself remained intact. Retention of syndecan-1-GFP and LAMP1-RFP markers at the release site confirmed that the endosome was not destroyed [13]. Dual-labeled polyplexes (FluoR-PEI and FITC-ODN) showed that polymer and cargo separated within the endosome at the moment of escape: the ODN was expelled while the polymer remnants lingered at the compartment before gradually fragmenting [13]. Only one to five polyplexes per cell contributed to productive delivery over a two-hour observation period, highlighting the extreme rarity of successful escape events [13]. These findings provide experimental evidence that escape and release are temporally coupled, with dissociation occurring in the endosome rather than in the cytosol. Yet this technique is low-throughput and cannot be readily scaled for carrier optimization.

### The Missing Measurement

Taken together, galectin recruitment quantifies how many endosomes are disrupted, SLEEQ quantifies how much carrier material reaches the cytosol, FRET reports whether the polyplex has dissociated, and live-cell imaging reveals the dynamics of a single escape event. But no existing assay measures the quantity of free, uncomplexed nucleic acid delivered to the cytosol from a single escape event. This is the measurement that the escape–release tradeoff framework demands. A carrier that disrupts many endosomes (high Gal8 recruitment) but fails to release its cargo (persistent FRET signal) would appear effective by one metric and ineffective by the other. The field needs an integrated assay, potentially combining a galectin reporter with a FRET-based unpacking sensor in the same cell, capable of resolving the complexation state of nucleic acid cargo at the moment of endosomal membrane disruption. Such a tool would enable direct measurement of what we have termed functional delivery efficiency: the fraction of internalized nucleic acid that reaches the cytosol in a translationally or catalytically competent state. Until this measurement becomes routine, the escape–release tradeoff will remain difficult to optimize experimentally.

## Translational Considerations: Why the Tradeoff Is Harder *In Vivo*

The escape–release balance described in the preceding sections has been characterized almost entirely in simplified model systems, typically immortalized cell lines, serum-free or low-serum conditions, and single-dose exposures. Several translational variables complicate this balance in ways that current *in vitro* optimization cannot fully anticipate.

### Protein Corona Reshapes the Electrostatic Landscape

Upon systemic administration, polymeric carriers rapidly acquire a protein corona that fundamentally alters the surface charge, hydrophobicity, and ligand accessibility governing both escape and release. Corona adsorption shields the cationic amines responsible for endosomal membrane destabilization, reducing escape capacity [162]. Simultaneously, anionic serum proteins can compete with nucleic acids for electrostatic binding sites on the polymer, potentially loosening condensation and triggering premature cargo release before the carrier reaches the endosome [163]. The tradeoff that was carefully tuned at the bench is thus re-set by the biological environment before the carrier ever encounters an endosome.

The composition of the corona is not random. It depends on the carrier's surface chemistry, size, charge, and PEG density, meaning that formulation changes intended to optimize escape or release will simultaneously change the corona profile and its downstream effects [164]. Furthermore, the corona evolves over time as the carrier moves from the bloodstream into tissues and through the endosomal pathway, with different protein populations adsorbing and desorbing at each stage [165, 166]. A carrier that retains adequate cationic charge for escape after initial corona formation in serum may lose that charge as the corona remodels during endosomal acidification, or it may gain new protein interactions that stabilize the polyplex against release. These dynamics are difficult to predict from static *in vitro* corona characterization and represent a significant gap between bench optimization and *in vivo* performance.

### Endosomal Acidification Varies Across Target Cells

The pH-responsive triggers discussed in Sect. "pH-responsive and charge-shifting polymers as escape–release coupling devices" assume a canonical acidification trajectory from pH 7.4 to approximately 5.0. In practice, endosomal maturation kinetics and terminal pH differ substantially across cell types. Macrophages acidify rapidly and reach lower terminal pH values than epithelial cells; hepatocytes differ from dendritic cells; tumor cells with upregulated proton pumps or altered lysosomal biogenesis present yet another profile [167]. A carrier with a pKₐ tuned for optimal escape at pH 6.2 in HeLa cells may not reach the same activation threshold in a target cell that acidifies more slowly, or may activate prematurely in a macrophage that acidifies faster.. This challenge is compounded in heterogeneous tissues such as solid tumors, where stromal, immune, and cancer cells coexist with distinct endosomal environments. Even within a single cell type, endosomal acidification is not uniform: individual endosomes within the same cell can mature at different rates depending on their cargo load, membrane composition, and position within the cell [168]. The consequence for carrier design is that a single pKₐ value cannot guarantee consistent escape timing across the target cell population, let alone across patients. This variability is rarely captured in standard *in vitro* screening, which typically uses a single cell line under uniform conditions.

### Tissue-specific Delivery Contexts Shift the Balance in Distinct Ways

Most escape–release strategies reviewed here have been developed and tested in tumor models, and the translational discussion so far has emphasized tumor-microenvironment triggers and tumor-cell endosomes. Other target tissues impose distinct constraints that a tumor-centric design will not address. Hepatocyte delivery through the asialoglycoprotein receptor is efficient, but Kupffer-cell uptake competes for delivered dose and imposes a macrophage-derived corona composition on any residual carrier reaching hepatocytes [169]. Pulmonary delivery must contend with the mucus barrier, the surfactant lining, and the rapid clearance by ciliated airway epithelium, each of which alters carrier surface chemistry before endocytosis begins [170]. Central nervous system delivery is constrained by the blood–brain barrier and by the low baseline endocytic activity of terminally differentiated neurons, restricting the accessible carrier pool to a small fraction of the systemic dose [171]. Immune-cell targets show divergent endocytic profiles: dendritic cells acidify rapidly and process endocytosed material efficiently for antigen presentation, whereas T cells have low baseline endocytic activity and rely on receptor engagement for productive uptake, an important constraint for CAR-T and other adoptive cell therapies increasingly delivered by nucleic acid carriers [172]. Skeletal muscle presents sarcolemmal barriers and extracellular matrix binding that slow both trafficking and release, though the slow turnover of muscle fibers can support durable expression if release timing is matched to the tissue's endosomal kinetics. Each of these targets shifts the escape–release balance not by changing the underlying tradeoff but by changing which side of the balance imposes the tightest constraint. Carriers optimized for tumor delivery will not translate one-for-one into hepatocyte, pulmonary, CNS, muscle, or immune-cell targets, and each application requires its own escape–release optimization.

### The PEG Dilemma Layers a Second Tradeoff on the First

PEGylation is essential for systemic circulation. It reduces opsonization, extends half-life, and improves biodistribution. However, PEG shields the same cationic and hydrophobic moieties required for endosomal membrane interaction, directly suppressing escape [91, 173]. Cleavable PEG strategies (acid-labile, MMP-cleavable, or reductively shed) attempt to resolve this by deshielding the carrier in the endosomal or tumor microenvironment, but these add another stimulus-responsive element whose trigger kinetics must be coordinated with the escape and release steps [174]. The system thus becomes a three-variable optimization: deshielding, escape, and release must occur in sequence within a narrow spatiotemporal window.

### Stimulus Heterogeneity Undermines Trigger-based Designs

The non-pH triggers evaluated in Sect. "Non-pH Stimulus-Responsive Strategies: Do Alternative Triggers Resolve the Tradeoff?" (GSH, ROS, esterases) vary not only between intracellular compartments but also between patients, disease stages, and tissue microenvironments. Tumor GSH concentrations can range from 2 to 40 mM across patients, and intratumoral ROS levels depend on immune infiltration, hypoxia, and treatment history [175, 176]. A disulfide-crosslinked carrier calibrated for 10 mM cytosolic GSH will behave very differently in a patient whose tumor cells contain 30 mM. Dual-trigger designs (Sect. "Dual-Trigger and Sequential-Trigger Designs: Toward Kinetic Alignment of Escape and Release") mitigate this somewhat by requiring two independent signals, but they also multiply the sources of variability.

This patient-level heterogeneity has implications beyond individual trigger concentrations. The overall redox balance of a tumor, the activity of its endosomal enzyme populations, and the rate at which its cells process endocytosed material all vary with tumor type, stage, prior treatment, and genetic background [177, 178]. Carriers optimized for the average tumor microenvironment will inevitably underperform in patients whose tumors fall outside that average, and current preclinical models rarely capture this diversity. The escape–release tradeoff is therefore not a single optimization problem with a single solution, but a distribution of problems whose parameters shift across the patient population.

Manufacturing complexity introduces additional variability. The multi-component, stimulus-responsive architectures discussed in Sects. "pH-responsive and charge-shifting polymers as escape–release coupling devices" through "Polymer Architecture and Formulation: Structural Approaches to Decoupling Escape from Release" (disulfide crosslinks, charge-shifting bonds, cleavable PEG, dual-trigger designs) are inherently more difficult to manufacture reproducibly than simple polycation systems. Batch-to-batch variation in crosslink density, PEG grafting efficiency, or trigger sensitivity could shift the escape–release balance in ways that are difficult to detect by standard physicochemical characterization (size, zeta potential, encapsulation efficiency) but that manifest as variable transfection efficiency or therapeutic response [24, 179]. As these systems move toward clinical translation, quality control metrics that specifically assess escape and release function, rather than only physical properties, will become essential.

These considerations do not invalidate the escape–release framework. They reinforce why it must be evaluated beyond the dish. The field's growing ability to measure escape (Sect. "Measuring Escape and Release: The Assay Gap") must be matched by efforts to measure it under conditions that approximate the biological complexity of the target tissue: in the presence of serum proteins, in disease-relevant primary cells, and ideally *in vivo*.

## Conclusion and Future Directions

This review has argued that endosomal escape and nucleic acid release are not independent optimization targets but are coupled through the same electrostatic and pH-dependent interactions that define polymeric carrier function. The cationic charge that enables a polymer to destabilize endosomal membranes simultaneously tightens its grip on the nucleic acid payload. This coupling is not a formulation inconvenience. It is a fundamental property of electrostatic complexation that persists across polymer classes, cargo types, and trigger chemistries. The strategies reviewed here each address part of the problem, but none fully resolve it.

Several gaps stand out as priorities for the field. First, the assay problem is urgent. No widely adopted method can simultaneously measure endosomal membrane disruption and the complexation state of the cargo at the moment of escape. Without this measurement, the tradeoff cannot be optimized rationally. We believe the most productive path forward is an integrated reporter system that combines a galectin-based membrane integrity sensor with a FRET-based unpacking readout in the same cell, enabling real-time resolution of whether cargo is free or polymer-bound as it enters the cytosol.

Second, escape and release should be evaluated under conditions that better approximate the *in vivo* environment, including full serum, disease-relevant primary cells, and physiologically appropriate cell densities. Protein corona, cell-type-dependent endosomal acidification, PEG shielding, and patient-level variability in redox environments all shift the escape–release balance in ways that standard *in vitro* screening does not capture. Evaluating escape and release in primary cells, in full serum, and in disease-relevant animal models is essential for translational progress.

Third, carrier design should be explicitly cargo-matched. The optimal polymer for pDNA over-condenses mRNA, and the polymer that releases siRNA efficiently cannot protect larger cargo. We think the field would benefit from routine multi-cargo screening early in the design process, testing each new polymer against at least two cargo types of different size to map the escape–release balance for each.

Fourth, and in our view most importantly, the most promising design direction is not stronger escape chemistry or more sophisticated triggers but systems in which the escape event itself initiates release. Charge-altering releasable transporters approach this through self-immolative degradation triggered by the pH shift upon cytosolic entry. Triblock architectures with spatially segregated domains offer a structural version of the same logic. The common principle is that release should not depend on a separate intracellular stimulus that may or may not be present at the right time and place. Instead, the physical act of leaving the endosome should be the trigger that liberates cargo. Engineering this escape-coupled release with sufficient kinetic precision to match the stochastic, low-frequency escape events reported across polyplex and LNP systems remains the central challenge [11, 29, 134]. An alternative approach worth exploring is whether tight electrostatic complexation is necessary at all. If a polymer and its nucleic acid cargo can be co-localized within the same nanoparticle without direct electrostatic binding, through compartmentalization, covalent conjugation, or physical confinement, the escape–release tradeoff as defined here would not apply. Developing non-complexation-based co-delivery architectures that still achieve endosomal escape may represent a path around the tradeoff rather than through it.

The escape–release tradeoff will not be eliminated by any single polymer chemistry. It is a consequence of the physics of polyelectrolyte complexation. But it can be managed through spatial segregation of functional domains, temporal coordination of state changes, and cargo-matched design, if the field develops the measurement tools and translational testing frameworks needed to optimize both sides of the balance simultaneously.

## Data Availability

Not applicable for this review article. No new data is presented.
